# Transcriptome changes in rice (Oryza sativa L.) in response to high night temperature stress at the early milky stage

**DOI:** 10.1186/s12864-015-1222-0

**Published:** 2015-01-23

**Authors:** Jiang-Lin Liao, Hui-Wen Zhou, Qi Peng, Ping-An Zhong, Hong-Yu Zhang, Chao He, Ying-Jin Huang

**Affiliations:** Key Laboratory of Crop Physiology, Ecology and Genetic Breeding (Jiangxi Agricultural University), Ministry of Education, Jiangxi Province, 330045 China; Key Laboratory of Agriculture responding to Climate Change (Jiangxi Agricultural University), Nanchang City, Jiangxi Province 330045 China

**Keywords:** Rice, Early milky stage, High night temperature, Transcriptomics, RNA-sequencing

## Abstract

**Background:**

Rice yield and quality are adversely affected by high temperatures, especially at night; high nighttime temperatures are more harmful to grain weight than high daytime temperatures. Unfortunately, global temperatures are consistently increasing at an alarming rate and the minimum nighttime temperature has increased three times as much as the corresponding maximum daytime temperature over the past few decades.

**Results:**

We analyzed the transcriptome profiles for rice grain from heat-tolerant and -sensitive lines in response to high night temperatures at the early milky stage using the Illumina Sequencing method. The analysis results for the sequencing data indicated that 35 transcripts showed different expressions between heat-tolerant and -sensitive rice, and RT-qPCR analyses confirmed the expression patterns of selected transcripts. Functional analysis of the differentially expressed transcripts indicated that 21 genes have functional annotation and their functions are mainly involved in oxidation-reduction (6 genes), metabolic (7 genes), transport (4 genes), transcript regulation (2 genes), defense response (1 gene) and photosynthetic (1 gene) processes. Based on the functional annotation of the differentially expressed genes, the possible process that regulates these differentially expressed transcripts in rice grain responding to high night temperature stress at the early milky stage was further analyzed. This analysis indicated that high night temperature stress disrupts electron transport in the mitochondria, which leads to changes in the concentration of hydrogen ions in the mitochondrial and cellular matrix and influences the activity of enzymes involved in TCA and its secondary metabolism in plant cells.

**Conclusions:**

Using Illumina sequencing technology, the differences between the transcriptomes of heat-tolerant and -sensitive rice lines in response to high night temperature stress at the early milky stage was described here for the first time. The candidate transcripts may provide genetic resources that may be useful in the improvement of heat-tolerant characters of rice. The model proposed here is based on differences in expression and transcription between two rice lines. In addition, the model may support future studies on the molecular mechanisms underlying plant responses to high night temperatures.

**Electronic supplementary material:**

The online version of this article (doi:10.1186/s12864-015-1222-0) contains supplementary material, which is available to authorized users.

## Background

The grain weight of rice (*Oryza sativa* L.) and other cereals is determined by the rate of grain growth and the duration of the growth period [1-3]. High temperature stress increases the growth rate of grain during the early ripening period and reduces the duration of growth, but the increase in the rate of grain growth fails to compensate for the reduction in the duration of grain growth, which ultimately results in a decrease in final grain weight [4,5]. Previous studies reported that high nighttime temperatures are more harmful to grain weight in rice and other crops than high daytime temperatures, because a deficiency of carbohydrates occurs in the leaves and culms due to increased respiration loss under high night temperatures [6,7]. Rice is one of the word’s major food sources, feeding more than half of the global population, and the world rice production must increase by 1% annually to meet the growing demand for food that will result from population growth and economic development. Unfortunately, the global surface temperature is rising consistently and the minimum nighttime temperature has increased approximately three times as much as the corresponding maximum daytime temperature over the past few decades [7].

It is well studied that the typical symptoms caused by high temperature stress during rice grain filling include an increased rice grain-filling rate, a low amylose content, an increased chalkiness degree and a poor milling quality [8-10]. Under high temperature stress, many of the starch synthase-related enzymes, such as granule-bound starch synthase (GBSS), soluble starch synthase (SSS) and starch debranching enzyme (DBE), were shown to be regulated by high temperatures [11-14]. A high temperature can also play a role in the down-regulation of the isoforms of the *SBE* genes (*SBEI, SBEIIb* and *SBEIII*) and up-regulation of the *SSS* isoforms (*SSSIIb, SSSIIc, SSSIIIb* and *SSSIVa*), and some starch-consuming genes, i.e., α-amylase (*Amy1A, Amy3D* and *Amy3E*) that cause low amylase contents in rice grain under high temperature stress [11,13]. Previous studies reported that the expression of *cyPPDKB* and *BEIIb* was down-regulated by high temperatures, which resulted in an increase in rice grain chalkiness, suggesting that *BEIIb* and *cyPPDKB* may be two of the candidate genes that play a critical role in grain chalkiness [6,15].

The development of cereal endosperm, including rice, goes through three major phases. In the first phase, the endosperm mainly undergoes cell division and differentiation to increase cell number. In the second phase, macromolecules accumulate to complete the endosperm cell enlargement, and in the third phase, the endosperm reaches maturity by undergoing starch and protein transformation [4,16,17]. It is well known that the damages and responding molecules of high temperature stress move from the second to the third phase of endosperm development, and the influence of high temperatures on endosperm development have been studied at enzyme/protein [18-20] and gene [10,13,15] levels. Previous studies also reported that the weight of rice grain was permanently damaged under high temperature stress at the early milky stage (8–15 days after flowering), and rice grain quality and weight could not be reversed, even if optimum temperature conditions were provided after the high temperature stress [21,22]. However, the molecular mechanism underlying these observations remains unclear.

The advent of next-generation high-throughput RNA sequencing technology has revolutionized transcriptome studies [23,24]. The technology is not only a powerful tool for discovering the full length of 5′ and 3′ untranslated regions, novel splice junctions, novel transcripts, alternative transcription start sites and rare transcripts [23,25,26], but can also detect and quantify gene expression using digital measurements, and is especially sensitive for low-expressed genes [27,28]. In addition, RNA-seq data show a high level of reproducibility in both technical and biological replicates [23,29].

In the present study, we used Illumina sequencing to compare the transcriptome differences between heat-tolerant and -sensitive rice lines responding to high night temperatures at the first phase of rice endosperm development (early milky stage). The goals of the current work were to identify potential candidate genes involved in the high night temperature response in rice. This may provide genetic resources that can be used for the improvement of heat-tolerant characters in rice. In addition, this work may provide a starting point for the elucidation of the molecular mechanism underlying the high night temperature stress response in rice.

## Results and discussion

### High night temperatures decreased grain plumpness (filled spikelets) of rice

It has been reported that plant growth and grain weight are strongly influenced by temperature [5,30]. In this study, rice grains were exposed to high night temperatures at the early milky stage (see methods), followed by normal growth conditions until maturity, and the grain plumpness of the heat-tolerant and -sensitive lines was determined and compared. The results showed that grain plumpness of the two lines decreased after 48 h (including two 10-h dark periods) of high night temperature stress, and the decrease ratio of grain plumpness in the heat-sensitive line decreased far more than in the heat-tolerant line [see Additional file 1]. This indicated that high night temperatures have different effects on heat-tolerant and -sensitive rice lines.

### Sequencing information

To compare the transcriptomes between the heat-tolerant and -sensitive lines responding to high temperature stress at the early milky stage, cDNA libraries were prepared from rice grain and subjected to RNA-Seq analysis on the Illumina HiSeq 2000 platform. A total of 1.34 billion raw reads were generated from the heat-tolerant and -sensitive lines.

A total of 1.25 billion (93.26%) high-quality 101-bp paired-end reads were selected for mapping to the Nipponbare reference genome by tophat [31]. The alignment results showed that 986 million (78.83%) of the high-quality reads were successfully mapped to the reference genome of *O. sativa* (Table 1). Of the mapped reads, 67.31-78.07% were mapped to exonic regions, 0.46-1.54% to intronic regions, 3.98-11.67% to intergenic regions and 17.29-25.33% to spliced regions (Table 2). These results suggest that a significant number of transcripts likely originated from alternative mRNA splicing or novel genes.

**Table 1:** **General information of sequencing reads and reads that mapped to the reference genome**

**Sample**	**Raw reads**	**High quality reads**		**Mapping to genome**	
		**Number**	**%**	**Number**	**%**
SC1	110,445,132	102,110,944	92.45	81,760,543	80.07
SC2	118,361,874	109,714,376	92.69	87,250,440	79.53
SC3	108,124,090	99,783,462	92.29	79,232,619	79.4
ST1	122,256,502	114,109,402	93.34	88,945,294	77.95
ST2	109,367,326	102,743,400	93.94	80,030,194	77.89
ST3	106,564,594	99,517,680	93.39	77,090,356	77.46
TC1	108,790,978	102,039,304	93.79	79,933,511	78.34
TC2	105,514,112	99,385,790	94.19	80,911,686	81.41
TC3	102,595,286	96,561,142	94.12	76,501,404	79.23
TT1	117,028,020	108,822,936	92.99	84,948,725	78.06
TT2	117,708,038	109,660,302	93.16	85,938,332	78.37
TT3	114,845,464	106,774,882	92.97	83,835,866	78.52
Total	1,341,601,416	1,251,223,620	93.26	986,378,970	78.83

“ST”, “SC”, “TT” and “TC” indicate the treatment and control of the heat-sensitive and -tolerant lines, respectively; “1”, “2” and “3” indicate three biological replicates.

**Table 2:** **Overview of reads that mapped to the genic and intergenic regions**

**Sample**	**Total MP**	**Exon**	**%**	**Intron**	**%**	**InterGenic**	**%**	**Spliced**	**%**
SC1	120,604,217	92,309,005	76.54	860,430	0.71	4,794,472	3.98	22,640,310	18.77
SC2	120,489,277	84,173,841	69.86	1,248,829	1.04	5,752,705	4.77	29,313,902	24.33
SC3	115,039,494	83,651,248	72.72	1,110,422	0.97	7,031,767	6.11	23,246,057	20.21
ST1	191,954,792	131,590,789	68.55	1,479,338	0.77	21,420,452	11.16	37,464,213	19.52
ST2	121,649,971	84,829,641	69.73	1,075,848	0.88	11,366,864	9.34	24,377,618	20.04
ST3	112,121,097	76,909,155	68.59	1,401,805	1.25	9,872,787	8.81	23,937,350	21.35
TC1	124,254,032	84,405,467	67.93	1,029,079	0.83	14,506,377	11.67	24,313,109	19.57
TC2	110,100,967	85,954,481	78.07	510,469	0.46	4,602,925	4.18	19,033,092	17.29
TC3	105,698,981	73,161,750	69.22	1,069,136	1.01	6,573,728	6.22	24,894,367	23.55
TT1	113,772,045	76,581,286	67.31	1,747,207	1.54	6,621,542	5.82	28,822,010	25.33
TT2	147,430,188	100,277,333	68.02	1,518,413	1.03	15,045,184	10.2	30,589,258	20.75
TT3	110,358,555	75,782,160	68.67	1,472,869	1.33	6,061,655	5.49	27,041,871	24.5
Total	1,493,473,616	1,049,626,156	70.28	14,523,845	0.97	113,650,458	7.61	315,673,157	21.14

“ST”, “SC”, “TT” and “TC” indicate the treatment and control of the heat-sensitive and -tolerant lines, respectively; “1”, “2” and “3” indicate three biological replicates.

The high-quality reads were then assembled using the Cufflinks program [32], and 35,272-42,377 assembly transcripts were obtained; the size of the assembly transcripts varied from 50 to 5000 bp [see Additional file 2], with an average size of 1,264-1,590 bp and N50 of 1,547-2,007 bp (Table 3). Of these assembled transcripts, 89.78-92.54% matched completely with the annotated rice genes, whereas 7.46-10.22% did not show any alignment hits to the rice genes that potentially originated from novel isoforms.

**Table 3:** **Assembly statistics**

**Sample**	**Number of transcripts**	**Average size (bp)**	**N50 (bp)**	**Known transcripts**		**Novel transcripts**	
				**Number (%)**	**Number of genes**	**Number (%)**	**Number of genes**
SC1	35,975	1,469.6	1,831	33,235 (92.38)	21,914	2,740 (7.62)	2194
SC2	39,333	1,507.2	1,899	36,022 (91.58)	23,528	3,311 (8.42)	2663
SC3	38,489	1,508.6	1,886	35,178 (91.40)	22,720	3,311 (8.60)	2641
ST1	40,989	1,394.4	1,802	37,256 (90.89)	23,998	3,733 (9.11)	2987
ST2	40,125	1,544.6	1,926	36,540 (91.07)	23,780	3,585 (8.93)	2837
ST3	40,972	1,584.6	1,983	37,026 (90.37)	23,759	3,946 (9.63)	3191
TC1	38,268	1,526.1	1,902	35,268 (92.16)	23,205	3,000 (7.84)	2398
TC2	35,272	1,264.2	1,547	32,639 (92.54)	22,862	2,633 (7.46)	2179
TC3	38,507	1,528.8	1,903	35,518 (92.24)	23,539	2,989 (7.76)	2397
TT1	42,377	1,590.2	2,007	38,090 (89.88)	24,419	4,287(10.12)	3458
TT2	40,585	1,399.2	1,823	36,939 (91.02)	23,603	3,646 (8.98)	2896
TT3	41,515	1,557.1	1,969	37,273 (89.78)	24,214	4,242(10.22)	3443

“ST”, “SC”, “TT” and “TC” indicate the treatment and control of the heat-sensitive and -tolerant lines, respectively; “1”, “2” and “3” indicate three biological replicates.

### Transcript analysis in response to high night temperature

Gene expression levels can be estimated from Illumina sequencing data based on the number of raw reads [33,34]. To identify differentially expressed transcripts (DEGs), the number of fragments mapped to each gene was calculated and normalized using fragments per kb per million fragments (FPKM) [32]. The FPKM value of each gene from the treatment vs. control was calculated according to the following criteria: p-value ≤ 0.05 and fold change (FC) log2 ≥ 1.0 [35,36]. The transcripts with FPKM values that fit the above criteria were considered for further analysis and are referred to as the high night temperature response transcripts (HTRTs) in the heat-tolerant or -sensitive lines in the present study.

**Figure 1:**
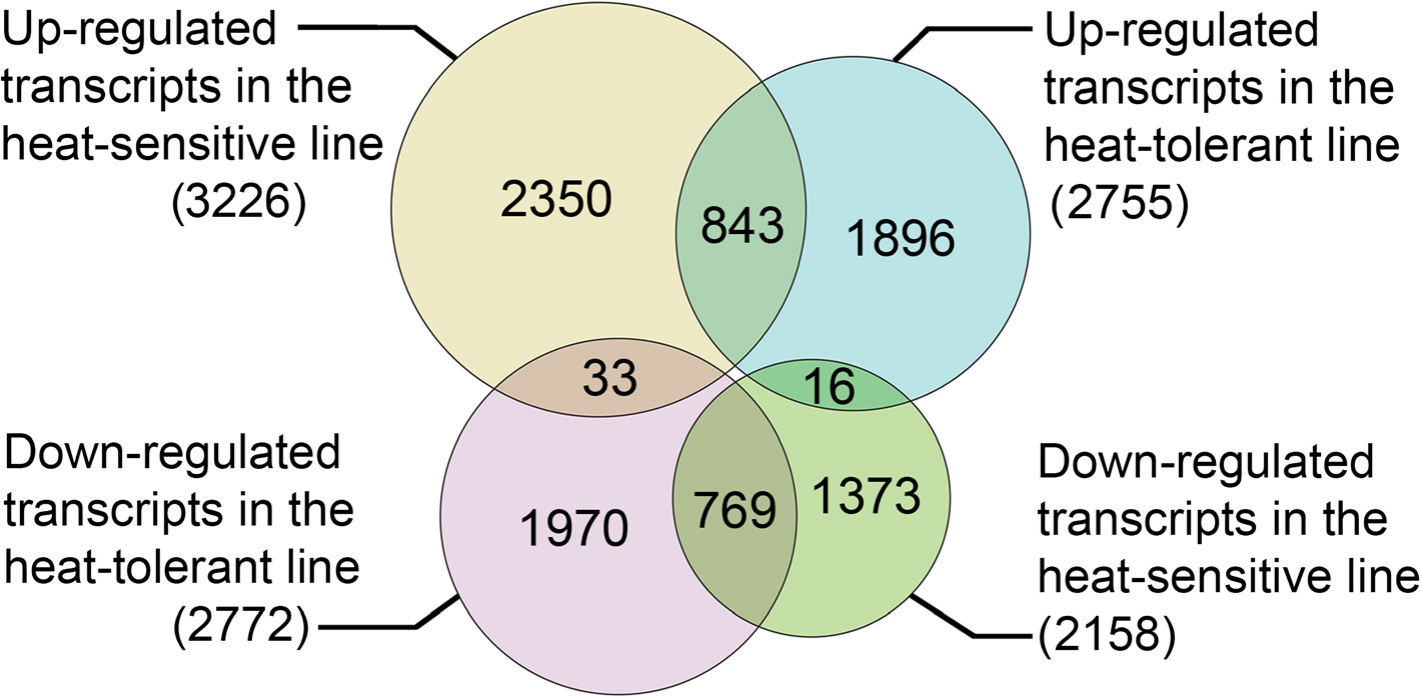
**The number of up- and down-regulated transcripts between the heat-sensitive and -tolerant lines.**

A total of 3,226 transcripts showed up-regulation and 2,158 transcripts showed down-regulation in the heat-sensitive line (Figure 1), whereas 2,755 transcripts showed up-regulation and 2,772 transcripts showed down-regulation in the heat-tolerant line, with a p-value ≤ 0.05 and FC log2 ≥ 1.0. Among the DEGs, 843 transcripts were up-regulated and 769 transcripts were down-regulated, both in the heat-tolerant and -sensitive rice lines. Interestingly, 33 transcripts were down-regulated in the heat-tolerant line but up-regulated in the heat-sensitive line, and 16 transcripts were up-regulated in the heat-tolerant line but down-regulated in the heat-sensitive line.

To assess the reproducibility of RNA-sequencing data, a cluster analysis of the 2-fold change values of the differentially expressed transcripts from three biological replicates was conducted, and the dendrogram analysis results [see Additional file 3] showed that the three biological replicates of the treatments and controls were clustered into one cluster, which suggested that the reproducibility of RNA-sequencing is reliable. To confirm the validity of the DEG data, RT-qPCR analysis was performed on 12 randomly selected genes, which indicated that these genes showed a similar gene expression to the pattern predicted by the RNA-sequencing method [see Additional file 4].

### GeneOntology (GO) analysis for HTRTs

**Figure 2:**
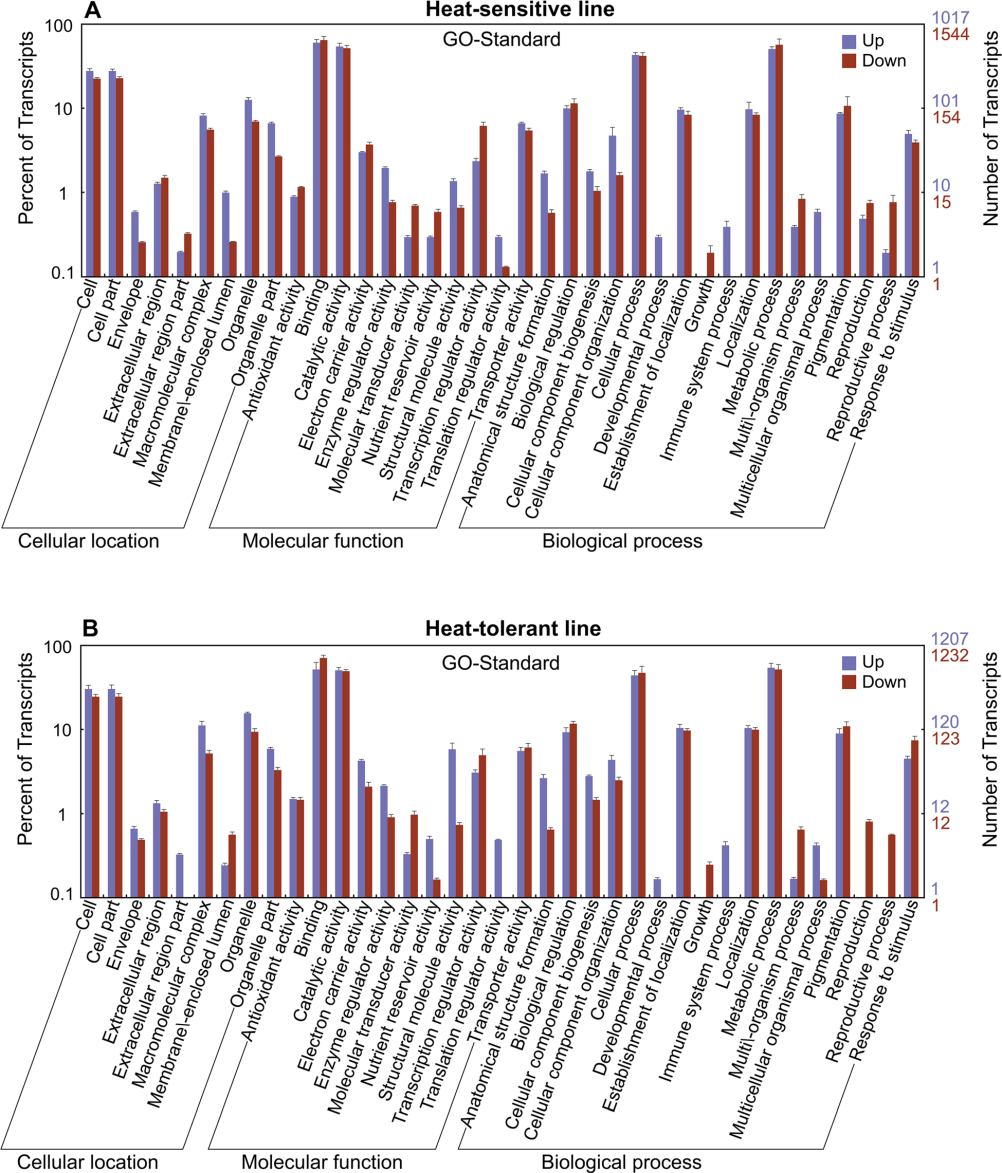
**Gene ontology classification of the unigenes from the heat-sensitive (A) and heat-tolerant (B) lines.**

Web Gene Ontology Annotation Plot (WEGO) software was used to perform the GO classifications and to draw the GO tree to classify the up- and down-regulated transcripts into putative functional groups for the heat-tolerant and -sensitive lines. A total of 9,155 and 9,011 transcripts were assigned GO terms for the heat-sensitive and -tolerant lines, respectively. Among the 9,155 transcripts from the heat-sensitive line (Figure 2A), there were 1,879 transcripts (890 showed up-regulation and 989 down-regulation) at the cellular level, 3,468 transcripts (1,346 showed up-regulation and 2,122 down-regulation) at the molecular level and 3,808 (1,506 showed up-regulation and 2,303 down-regulation) transcripts at the biological level. Among the 9,011 transcripts from the heat-sensitive line (Figure 2B), there were 2,019 transcripts (1,159 showed up-regulation and 860 down-regulation) at the cellular level, 3,228 transcripts (1,529 showed up-regulation and 1,699 down-regulation) at the molecular level and 3,764 transcripts (1,843 showed up-regulation and 1,921 down-regulation) at the biological level (Figure 2B). Within the cellular component category, transcripts that corresponded to cell (12,559) and cell organelles (12,559) were the most abundant. Binding (31,859) and catalytic activities (25,935) were the most abundant groups within the molecular functional category. A total of 15 GO functional groups were assigned to the biological processes category, among which metabolic processes (24,958) and cellular processes (22,198) were the most highly represented.

A comparison of the GO trees of the DEGs between the heat-tolerant and -sensitive lines revealed a higher number of down-regulated transcripts involved in extracellular regions, and a higher number of up-regulated transcripts involved in membrane-enclosed lumens at the cellular location category in the heat-sensitive line compared with the heat-tolerant line. This indicates that a high night temperature disrupts the cell-cell interactions and plasmalemma functioning in plant cells, and these effects exhibited different patterns in the heat-tolerant and -sensitive lines. In the molecular functional category, there was a higher number of down-regulated transcripts involved in nutrient reservoir and translation regulator activities in the heat-sensitive line compared with the heat-tolerant line. For the biological processes category, there was a higher number of up-regulated transcripts involved in reproduction and reproductive processes in the heat-sensitive line compared with the heat-tolerant line. This suggests that a high night temperature affects the reproduction of the plant cell, and the effects were different between the heat-sensitive and heat–tolerant lines.

### DEGs of the HTRTs between the heat-tolerant vs. heat-sensitive lines

To identify differentially expressed HTRTs between heat-tolerant and -sensitive lines, the changes in the expression levels between the 5,384 DEGs (3,226 showed up-regulation and 2,158 showed down-regulation) from the heat-sensitive line and the 5,527 DEGs (2,755 showed up-regulation and 2,772 down-regulation) from the heat-tolerant line were further compared. The transcripts that showed a Relative Fold Change (RFC) ≥ 2.0 were selected for downstream analysis. A total of 35 transcripts showed different expression between the heat-tolerant and -sensitive rice, and the base sequences and FPKM values of the 35 transcripts are presented in Additional file 5. The results suggested that a high night temperature triggered thousand of transcripts to differentially express in the heat-tolerant and -sensitive lines, but only 35 transcripts showed a significant difference (RFC ≥ 2.0) in gene expression between the two lines. We suggest that these findings result from the similar genomics of the two rice materials (see Method-Plant materials) and the results provided a solid basis for our further study on selecting and cloning heat-resistant gene(s) in rice.

**Figure 3:**
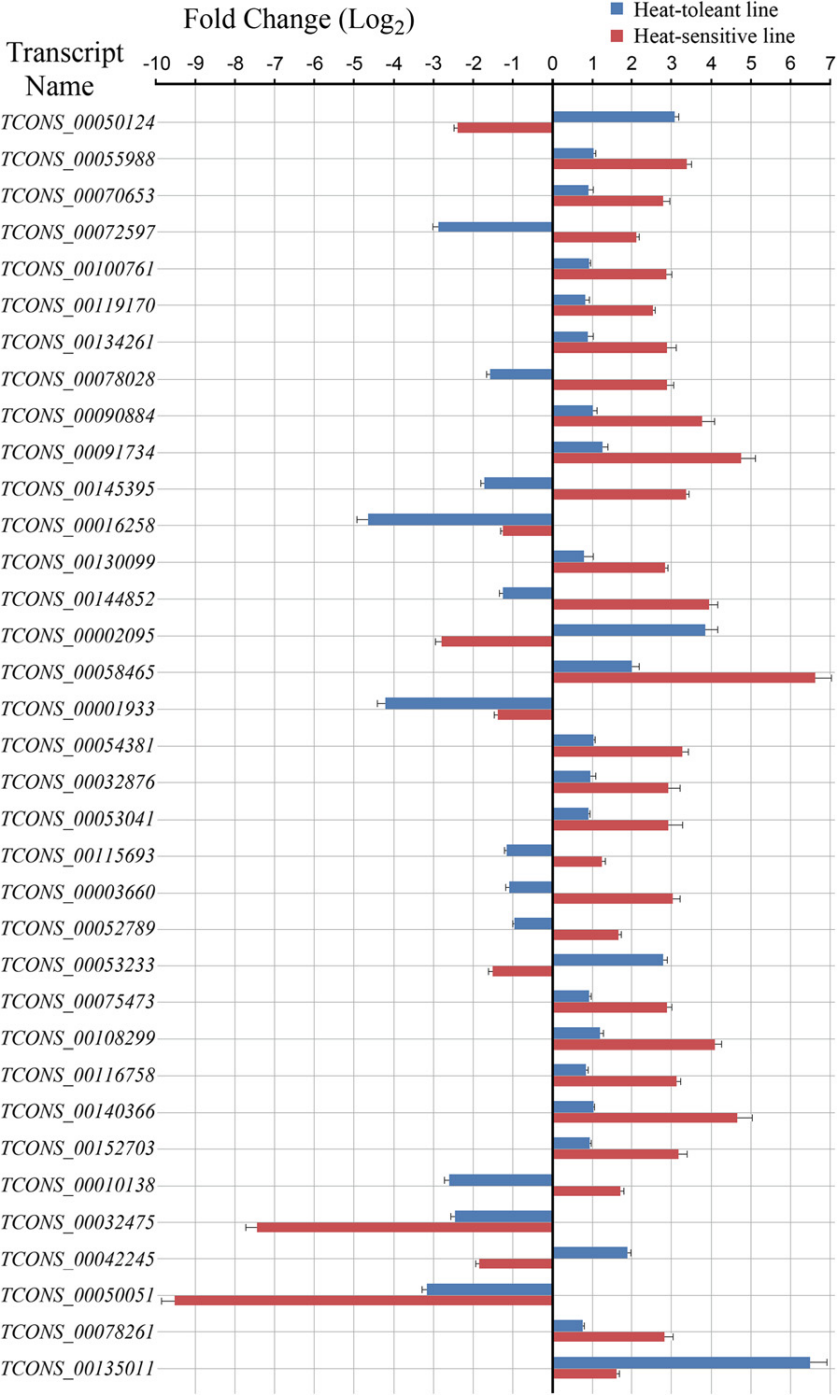
**The expression patterns of the different HTRTs between the heat-tolerant and -sensitive lines.** HTRTs: high night temperature response transcripts. Bar chart on the left of the x-axis denotes the down-regulated transcripts and bar chart on the right of the x-axis denotes up-regulated transcripts.

Of the 35 differentially expressed transcripts, 4 were down-regulated and 19 were up-regulated in both the heat-tolerant and -sensitive lines. In addition, 4 transcripts were up-regulated in the heat-tolerant line but down-regulated in the heat-sensitive line, and the other 8 transcripts were down-regulated in the heat-tolerant line but up-regulated in the heat-sensitive line (Figure 3).

### Functional annotation of the DEGs of the HTRTs

To annotate the 35 DEGs of the HTRTs, assembled transcript sequences were blasted to the National Center for Biotechnology Information (NCBI) website, against the non-redundant (nr) protein database with a cut-off e-value of 10^−5^. Among the 35 DEGs, 21 were functionally annotated based on the nr-NCBI database (Table 4). Based on the functional annotation, the 21 unigenes were classified into six groups: oxidation-reduction (6 genes), metabolic (7 genes), transport (4 genes), transcript regulation (2 genes), defense response (1 gene) and photosynthetic (1 gene) processes. Of the 14 HTRTs [see Additional file 6], 8 were found to have homologous genes but without functional annotation, whereas six HTRTs did not show any hits in the nr-NCBI database.

**Table 4:** **The differentially expressed HTRTs between heat-tolerant and -sensitive rice lines with their corresponding reference genes, functional annotations and protein functions**

**Transcripts name**	**Size (bp)**	**Reference gene in GenBank**	**Interpro hits**		**Molecular function**
			**ID**	**Annotation**	
Oxidation					
*TCONS_00050124*	1767	OJ1115_B01.19	IPR002401	Cytochrome P450, E-class, group I	Oxidoreductase activity; acting on paired donors, with incorporation or reduction of molecular oxygen
*TCONS_00134261*	1808	P0025D09.112	IPR002401	Cytochrome P450, E-class, group I	Oxidoreductase activity; acting on paired donors, with incorporation or reduction of molecular oxygen
*TCONS_00070653*	1973	OS03G0760000	IPR002401	Cytochrome P450, E-class, group I	Oxidoreductase activity; acting on paired donors, with incorporation or reduction of molecular oxygen
*TCONS_00055988*	3243	OJ1353_F08.4	IPR000572	Oxidoreductase, molybdopterin- binding domain	Electron carrier activity; catalyses oxidation of nitrate to nitrite, using cytochrome c as the physiological electron acceptor
*TCONS_00100761*	2437	OSJNBA0083N12.10	IPR002680	Alternative oxidase	Alternative oxidase activity; used as a second terminal oxidase in the mitochondria, electrons are transferred directly from reduced ubiquinol to oxygen forming water
*TCONS_00072597*	1096	OS03G0693900	IPR001929	Germin	Manganese ion binding; act as oxalate oxidases or as superoxide dismutase
Transportation					
*TCONS_00130099*	2072	P0567H04.25	IPR003663	Sugar/inositol transporter	Substrate-specific transmembrane transporter activity; binding and transport of various carbohydrates, organic alcohols, and acids
*TCONS_00144852*	1741	P0498H04.19	IPR003480	Transferase	Transferase activity; transferring acyl groups other than amino-acyl groups
*TCONS_00053041*	1016	OJ1004_A05.35	IPR000566	Lipocalin/cytosolic fatty- acid binding domain	Transport small hydrophobic molecules such as steroids, bilins, retinoids
*TCONS_00001933*	1107	P0432B10.23	IPR016140	Bifunctional inhibitor/plant lipid transfer protein/ seed storage helical domain	A transporter of fatty acids or fatty acid derivatives (e.g. hydroxy-fatty acids, acyl-CoA) necessary during the formation of cutin layers or during lipid mobilization in germinating seedlings
Metabolism					
*TCONS_00016258*	2627	OS01G0150100	IPR001330	Prenyltransferase/squalene oxidase	Catalytic activity; catalyzes the cyclization of (S)-2,3- epoxysqualene to lanosterol, the initial precursor of steroid hormones and vitamin D
*TCONS_00119170*	794	P0592E11.19	IPR015797	NUDIX hydrolase domain-like	Hydrolase activity; catalyze the hydrolysis of a variety of nucleo-side diphosphate derivatives
*TCONS_00078028*	1390	OS03G0790500	IPR013094	Alpha/ beta hydrolase fold-3	Hydrolase activity; a number of hydrolytic enzymes of widely differing phylogenetic origin and catalytic function
*TCONS_00090884*	1102	OSJNBA0041A02.26	IPR000757	Glycoside hydrolase, family 16 site	Hydrolase activity; hydrolyzing O-glycosyl compounds
*TCONS_00091734*	2019	OSJNBA0064H22.6	IPR010107	Glutamate decarboxylase	Glutamate decarboxylase activity; catalyze the irreversible alpha- decarboxylation of L-glutamate to gamma-amino-butyrate (GABA).
*TCONS_00145395*	1856	OJ1368_G08.14	IPR010977	Aromatic-L-amino-acid decarboxylase	Carboxy-lyase activity; catalyses the decarboxylation of tryptophan to tryptamine
*TCONS_00054381*	2231	P0705A04.34	IPR001461	Aspartic peptidase	Aspartic-type endopeptidase activity; encode for an aspartyl protease which is a homodimer of a chain of about 95 to 125 amino acids.
Transcription					
T*CONS_00002095*	1776	NCRNA_5217	IPR001584	Integrase, catalytic core	Nucleic acid binding
*TCONS_00058465*	1242	DREB1G	IPR001471	AP2/ERF domain	Sequence-specific DNA binding transcription factor activity
Photosynthesis					
*TCONS_00115693*	877	P0644A02.28	IPR003245	Plastocyanin-like	Electron carrier activity; exchange electrons with cytochrome c6
Defense					
*TCONS_00032876*	2297	OS11G0249000	IPR000767	Disease resistance protein	Plant defense response to pathogens

### Expression patterns of the unigenes

Electron transfer is a fundamental process in all living organisms. In plants, most electron relay systems lie in the mitochondrial respiratory chain and in the photosystems in chloroplasts. In the present study, five unigenes (*TCONS_00050124*, *TCONS_00134261*, *TCONS_00070653*, *TCONS_00055988* and *TCONS_00100761*) involved in electron transfer in the mitochondrial respiratory chain and one (*TCONS_00115693*) in the photosystem were found to be differentially expressed in the heat-tolerant and -sensitive lines after high temperature exposure. Among the five unigenes involved in the mitochondrial respiratory chain, the molecular functions of three unigenes (*TCONS_00050124*, *TCONS_00134261* and *TCONS_00070653*) was related to cytochrome P450 activity. Cytochrome P450, which catalyzes electron transfer through the pathway from NADPH to flavin adenine dinucleotide (FAD), then to flavin mononucleotide (FMN), and finally to heme groups containing redox partners [37,38], is also reported to be involved in reproductive development and act as a maternal regulator of seed size by stimulating integument cell proliferation in *Arabidopsis* [39-41]. One unigene (*TCONS_00055988*) with an annotation of molybdopterin-binding domain protein, which is known as the molybdopterin cofactor, is required for eukaryotic oxidoreductases to catalyze the reduction of nitrate to nitrite in a single polypeptide electron transport chain with electron flow from NAD(P)H-FAD-cytochrome b5-molybdopterin-NO(3) [42]. Another unigene (*TCONS_00100761*) is alternative oxidase, which is a second terminal oxidase in the mitochondria, and which transfers electrons directly from reduced ubiquinol to oxygen forming water [43].

**Figure 4:**
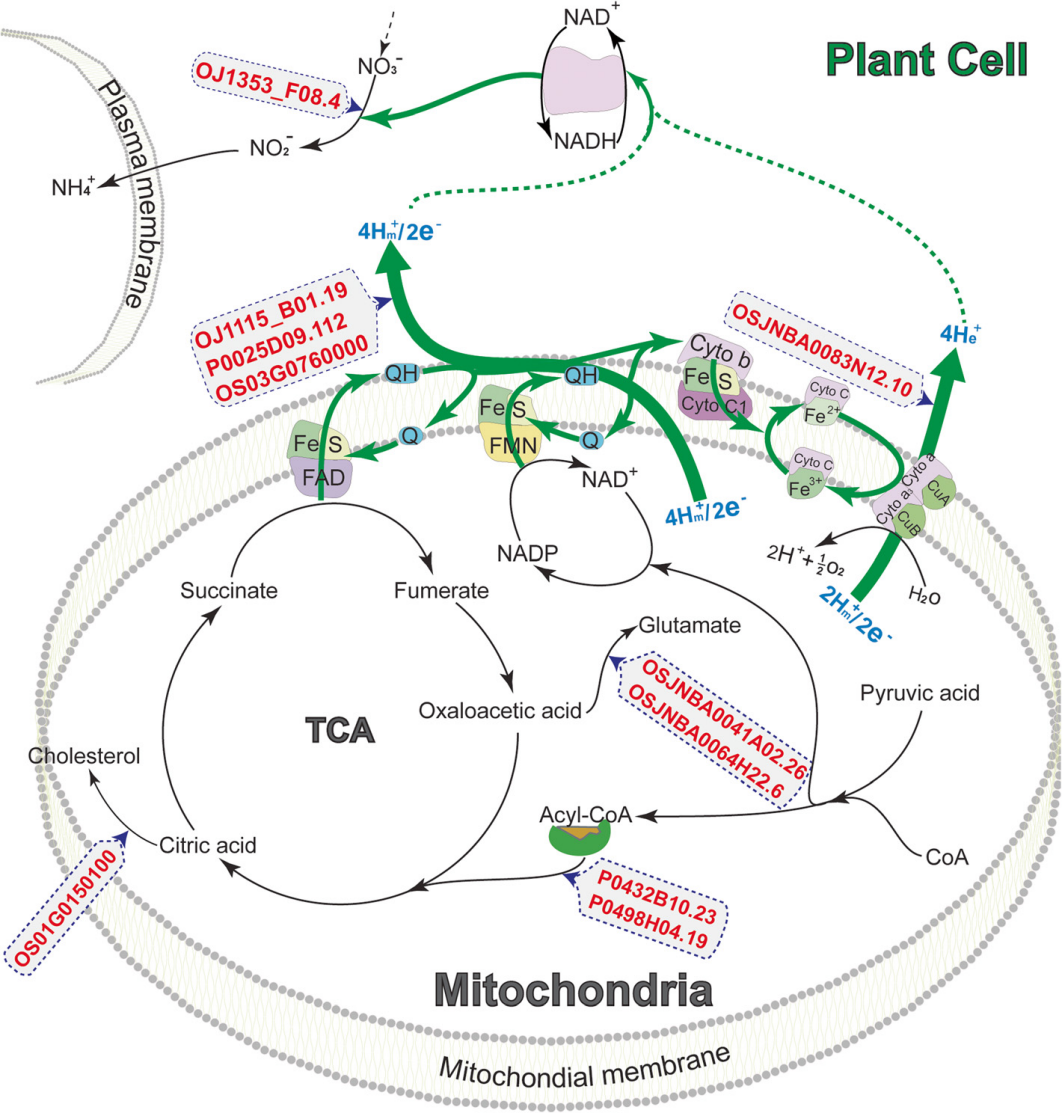
**Possible regulating processes of the differentially expressed HTRTs in rice grain cells in response to high night temperatures at the early milky stage.** Blue arrow indicates the electron transport orientation; Red words indicate the differentially expressed HTRTs, and the arrow pointing to the box indicates the regulated pathway. TCA: tricarboxylic acid cycle; FAD: flavin adenine dinucleotide; FMN: flavin mononucleotide; Q in the electron transport chain indicates ubiquinone (coenzyme Q, CoQ); Cyto: cytochrome; NO_3_^−^: nitrate; NO_2_^−^: nitrite.

Comparing the gene expression patterns between the heat-tolerant and -sensitive lines, four unigenes (*TCONS_00134261*, *TCONS_00070653*, *TCONS_00055988* and *TCONS_00100761*) were up-regulated in both the heat-tolerant and -sensitive lines, but up-regulation was more pronounced in the heat-sensitive than in the heat-tolerant line (Figure 4). Interestingly, one of the cytochrome P450 unigenes (*TCONS_00050124*) showed a 3-fold up-regulation in the heat-sensitive line but more than a 2-fold down-regulation in the heat-tolerant line. The unigene (*TCONS_00115693*), which is involved in electron transfer in the photosystem, showed up-regulation in the heat-sensitive line but down-regulation in the heat-tolerant line. It was reported that the overexpression of the P450 cytochrome gene *CYP78A* in *Arabidopsis* produced a general growth phenotype such as reduced fertility, and the knockout line of the P450 cytochrome gene *CYP78A5* showed a decreased cell number but not a decreased cell size [40]. In our study, the P450 cytochrome unigenes in the heat-sensitive line pronounced more up-regulation than in the heat-tolerant line, but the heat-sensitive line showed lower grain plumpness compared with the heat-tolerant line, which suggests that a higher expression level of the P450 unigenes would restrict grain plumpness in rice. Collectively, these results suggest that the electron transfer chains of mitochondrial respiration and the photosystems were influenced by high night temperatures, but there were differences in the damage patterns between the heat-tolerant and -sensitive lines.

### Amino acid metabolism was influenced by high night temperature

Some amino acids or metabolic intermediates play an important role in plant growth and developmental processes, as well as in the resistance of plants to a variety of stresses, including both biotic and abiotic stresses [44]. For example, Brassinosteroids, a class of plant-specific steroidal hormones, were reported to influence grain yield, plant height, leaf angle, grain size and tiller number [45]. Gamma-aminobutyric acid (GABA), a non-protein amino acid converted by L-glutamate, was reported to be related to a blast pathogen [46] and salt stress [47,48]. Aspartic proteases,which are distributed in all living organisms, were reported to be involved in pollen germination and growth [49], disease resistance [50], drought and abscisic acid (ABA)-dependent signal transduction [51]. In the present study, unigenes involved in the synthesis of steroid hormones (*TCONS_00016258*), glycosyl (*TCONS_00090884*), glutamate (*TCONS_00091734*) and aspartic peptidase (*TCONS_00054381*) were found to have different expressions in the heat-tolerant and -sensitive lines after high temperature stress.

With respect to the expression patterns, the unigenes involved in glycosyl, glutamate and aspartic peptidase synthesis showed up-regulation in both the heat-tolerant and -sensitive lines, but up-regulation was more pronounced in the heat-sensitive line. A previous study reported that the aspartic protease *ASPG1* gene in *Arabidopsis* was significantly up-regulated and triggered the expression of downstream targets in ABA signal transduction under drought conditions, which in turn promoted reactive oxygen species (ROS) production and caused oxidative damage in plant cells [51]. In the present study, the aspartic peptidase unigene *TCONS_00054381* had a more pronounced up-regulation in the heat-sensitive than in the heat-tolerant line, which suggests that oxidative damage was more serious and led to low grain plumpness in the heat-sensitive line compared with the heat-tolerant line.

In plants, steroid hormones are involved in regulating gene expression to regulate plant growth and developmental processes such as cell elongation, photomorphogenesis and stress responses [44]. In the present study, the transcript (*TCONS_00016258*) involved in steroid hormone synthesis was down-regulated in both the heat-tolerant and -sensitive lines. The transcript showed more than 4-fold down-regulation in the heat-tolerant line but less than 2-fold down-regulation in the heat-sensitive line. However, the grain plumpness of the heat-sensitive line was lower than that of the heat-tolerant line under high night temperature stress, which suggests that reduced steroid hormones in plant cells may be beneficial for rice grain plumpness.

### Transmembrane transport of metabolic intermediates was influenced by high night temperature

Transport of solutes and intermediates across cellular plasma membranes is necessary for complex metabolism. In this study, four unigenes involved in the transmembrane transport of sugar (*TCONS_00130099*), acyl (*TCONS_00144852*), hydrophobic molecules (*TCONS_00053041*) and fatty acids (*TCONS_00001933*) were found to be influenced by high night temperature stress.

For the expression pattern, the fatty acids transporter related unigene (*TCONS_00001933*) was down-regulated in both the heat-tolerant and -sensitive lines, and the unigene showed more than 3.5-fold greater down-regulation in the heat-tolerant line compared with the heat-sensitive line after high night temperature stress. The sugar transporter (*TCONS_00130099*) and hydrophobic molecule transporter (*TCONS_00053041*) showed higher expression in the heat-tolerant and -sensitive rice lines than in the controls, but up-regulation was more pronounced in the heat-sensitive line. In addition, the acyl transporter related unigene (*TCONS_00144852*) showed a 4-fold up-regulation in the heat-sensitive line but was down-regulated in the heat-tolerant line under high night temperature stress (Figure 4). The results of the gene expression patterns suggest that night warming stimulates dark respiration and leads to a depletion of carbohydrates in plant cells [52]. However, there was greater depletion of carbohydrates in the heat-sensitive line than in the heat-tolerant line, and this resulted in a low grain plumpness in the heat-sensitive line.

### Hydrolysis and decarboxylization in rice cells were influenced by high temperature stress

Some Nudix hydrolases are reported to play regulatory roles in preventing accumulation of reactive nucleoside diphosphate derivatives, cell signaling molecules or metabolic intermediates by diverting them to metabolic pathways to maintain physiological homeostasis by sensing and modulating the levels of their substrates [53]. In the present study, three unigenes (*TCONS_00119170*, *TCONS_00078028* and *TCONS_00090884*) involved in hydrolysis and two unigenes (*TCONS_00091734* and *TCONS_00145395*) involved in decarboxylization were influenced by high night temperature stress. Comparing the expressed patterns of their parallel controls, the NUDIX hydrolase related unigene (*TCONS_00119170*) and glycoside hydrolase related unigene (*TCONS_00090884*) involved in hydrolysis, and the glutamate decarboxylase related unigene (*TCONS_00091734*) involved in decarboxylization showed up-regulation in both the heat-sensitive and heat-tolerant lines. In addition, the expression of the transcript was 3.5-fold higher in the heat-sensitive line compared with the heat-tolerant line. In contrast, the Alpha/beta hydrolase related unigene (*TCONS_00078028*) involved in hydrolysis and Aromatic-L-amino-acid decarboxylase related unigene (*TCONS_00145395*) involved in decarboxylization were up-regulated in the heat-sensitive line but down-regulated in the heat-tolerant line. The expression patterns of the genes related to hydrolysis suggest that the disturbance of physiological homeostasis in the heat-sensitive line was greater than in the heat-tolerant line.

### Unknown transcripts may play an important role in rice responding to high night temperature stress

Fourteen transcripts without functional annotation exhibited differentially expressed patterns between the heat-sensitive and heat-tolerant lines. Interestingly, the unigene *TCONS_00135011* showed more than 6-fold greater up-regulation compared with its control in the heat-tolerant line but less than 2-fold greater up-regulation in the heat-sensitive line. The unigene *TCONS_00050051* was down-regulated more than 9-fold compared with its control in the heat-sensitive line but only down-regulated 3-fold in the heat-tolerant line. Further, the unigene *TCONS_00032475* was down-regulated more than 7-fold compared with its control in the heat-sensitive line but was only down-regulated 2.5-fold in the heat-tolerant line. We suggest that these three unigenes may play an important role in rice response to high night temperature stress.

### Proposed model that regulates the expression of differentially expressed transcripts during high temperature stress in rice at the early milky stage

Based on the functions of the differentially expressed HTRTs, we proposed a possible mechanism under which these unigenes function in response to heat shock in the plant cell (Figure 4). Initially, heat shock influenced the enzyme (*TCONS_00050124*, *TCONS_00134261*, *TCONS_00070653* and *TCONS_00100761*) activities involved in electron transport under normal conditions, and disrupted electron transport from the intramembrane to the extramembrane in mitochondria, including the electron transport chains from NADP-NAD+ to FMN, FAD to ubiquinol-Q, and ubiquinol-Q to oxygen forming water, which led to changes in the concentration of the hydrogen ion in the mitochondrial and cellular matrix. In the mitochondrial matrix, the activities of the transporter for acyl-CoA (*TCONS_00001933* and *TCONS_00144852*), glycoside hydrolase activities (*TCONS_00090884* and *TCONS_00091734*) and glutamate decarboxylase activities (*TCONS_00016258*) were influenced by changes in the concentration of the hydrogen ion, which influenced the metabolism of oxaloacetic acid and citric acid, and further affected the metabolism of succinate to fumarate in the tricarboxylic acid cycle (TCA). The disruption of the metabolism of succinate to fumarate may result in feedback regulation of electron transport from the TCA to the mitochondrial membrane. In the cellular matrix, the electron transport chain from NO_3_^−^ to NH_4_^+^ was also influenced by changes in the hydrogen ion concentration. According to our proposed model and the different expression patterns of the unigenes related to electron transfer, oxidation, hydrolase activities, depletion of carbohydrates and the production of metabolic intermediates involved in physiological homeostasis, it suggested that a high night temperature resulted in a more pronounced up-regulation and increased depletion of carbohydrates in the heat-sensitive line than in the heat-tolerant line, which led to lower carbohydrate accumulation and resulted in a low grain plumpness in the heat-sensitive line.

Comparing our previous results from transcriptome [54] and proteomics [20] studies in rice responding to high temperature stress (at daytime and nighttime), we suggest that high temperature may mainly affect the expression of genes related to oxidation, while a high night temperature could exert a major influence on the expression of genes related to the electron transport from the intramembrane to the extramembrane of mitochondria in rice grains at the early milky stage.

## Conclusions

Using Illumina sequencing technology, the differences in the transcriptomes of heat-sensitive and -tolerant rice lines in response to high night temperature stress at the early milky stage are described here for the first time. The candidate transcripts may provide genetic resources for the improvement of heat-tolerant characters in rice. The proposed model, which is based on transcription expression differences between two rice lines, may facilitate future studies of the molecular mechanisms underlying high night temperature responses in plants.

## Methods

### Plant materials

Two rice lines, heat-tolerant XN0437T and heat-sensitive XN0437S, which originated from RIL inbred populations in our previous study, were used as the plant materials in this study. These lines showed only 1.80% genomic polymorphism based on the 887 SSR markers and showed significant differences in grain weight when exposed to high temperatures during the grain ripening period [55].

### Growth conditions and temperature treatments

Rice was cultured using a tub-planting method described previously [20,53]. In order to ensure that only uniformly developed samples were used for analysis, rice ears with the same heading date were labelled, and then florets on the labelled ears with the same flowering date were further labelled.

On the 8^th^ day after the labeled florets flowered, plants with the same label were transferred to chambers and maintained at a temperature of 38.0 ± 0.5°C (treatment) or 25.0 ± 0.5°C (control) for the dark period (10 h), and 26.0 ± 0.5°C (both treatment and control) for the light period (14 h). Three biological replicates of the temperature treatments were grown under the same conditions.

After 48 h of treatment, samples containing 45 grains with labels from the same region (middle to bottom part) of labelled ears were harvested, packed in aluminum foil, and flash-frozen in liquid nitrogen until further use. A total of 12 rice grain samples were harvested, i.e., controls (TC1, TC2 and TC3) and treatments (TT1, TT2 and TT3) of the three biological replicates of the heat-tolerant line, and controls (SC1, SC2 and SC3) and treatments (ST1, ST2 and ST3) of the three biological replicates of the heat-sensitive line.

To determine the effect of a high night temperature on the heat-tolerant and -sensitive lines, plants were moved to natural growth conditions until maturity, when grain plumpness (GP) was measured. GP was calculated using the formula GP (%) =100% × GWt/ GWc; GWt and GWc represent the weight of 1000 grains from the treatment and control, respectively. The three biological replicates of the temperature treatments were grown at different time periods under the same growth conditions.

### RNA extraction and mRNA library construction

Total RNA was isolated from 12 rice panicle samples, including the controls, from three biological replicates (SC1, SC2 and SC3). These three biological replicates (ST1, ST2 and ST3) from the heat-sensitive line, the controls of the three biological replicates (TC1, TC2 and TC3), and the three biological replicates (TT1, TT2 and TT3) from the heat-tolerant line were treated using the RNeasy Plus Plant Kit (Qiagen, Germany), and the contaminated genomic DNA was removed using the RNeasy® MinElute® Cleanup Kit (Qiagen, Germany) following the manufacturer’s instructions. The RNA quality was checked using Bioanalyzer 2100 (Agilent, CA) with a minimum RNA Integrity Number (RIN) value of 8.5. Sequencing libraries were prepared using the TruSeq RNA Sample Preparation Kit (Illumina, CA) according to the manufacturer’s protocol as follows. Briefly, the poly (A)^+^RNA was isolated from 4 μg of the pooled total RNA from the 12 samples (SC1, SC2, SC3, ST1, ST2 ST3, TC1, TC2 TC3, TT1, TT2 and TT3) using Dynal oligo(dT)_25_ beads, and fragmented using divalent cations at 95°C. The fragmented RNA fragments were reverse transcribed into first strand cDNA using reverse transcriptase and random hexamers. Following the second strand cDNA synthesis and adaptor ligation, 300–350 bp cDNA fragments were isolated using gel electrophoresis and amplified by 12 cycles of PCR to produce the library for cluster generation and sequencing.

### Illumina sequencing

The libraries (3 per lane) were loaded onto an Illumina HiSeq™ 2000 instrument and subjected to 100 cycles of paired-end (2 × 101 bp) sequencing that generated 10 Gb per sample. For the multiplexing sequencing, 100 cycles of single read 1 was used to sequence the RNA, followed by 7 cycles of index identification and 100 cycles of single read 2. Primary data analysis and base calling were performed using the Illumina instrument software (Illumina’s Consensus Assessment of Sequence and Variation, CASAVA).

### Reads filtration and assembly

Before assembly, adaptor sequences, empty reads, low quality sequences with ‘N’ percentage over 5% and those containing more than 20% bases with a Q-value < 20 were removed using the Perl program written according to the custom method of Program editing. The retained high quality reads were mapped to the Nipponbare reference genome [56] by tophat [31], and then assembled with Cufflinks [32].

### Functional annotation of transcripts

Information was obtained regarding functional annotation, including protein functional annotation, COG functional annotation, and GO functional annotation of unigenes. Unigene sequences were first submitted to the protein databases for alignment and comparison by BLASTX algorithms with a significant threshold e-value ≤ 1e-5, from the Nr-NCBI, SWISS-PROT, COG and KEGG databases. In addition, unigenes were aligned by Blastn to nucleotide databases nt (e-value ≤ 1e-5) to retrieve functional annotations based on proteins with the highest sequence similarity to the given unigenes. We used the ESTScan program to predict the CDS and orientation of the assembled transcripts. With nr-NCBI annotation, the Blast2GO program was used to classify unigenes to GO terms based on molecular function, biological processes and cellular components [57].

### Validation of RNA-Seq by RT-qPCR

RT-qPCR was performed to verify the expression patterns revealed by the RNA-seq study. Total RNA extraction and generation of first-strand cDNA generation were performed according to the protocol used for the RNA-seq study mentioned above. Twelve unigenes were selected based on their possible functions and expression patterns in RNA-Seq. Primer sequences [see Additional file 7] were designed on exon-exon boundaries using the online primer software package primer-BLAST in NCBI. RT-qPCR was performed in an ABI 7500 FAST Real-Time PCR System using a protocol described in a previous study [20,53]. The amplicons were used for melting curve analysis to confirm the specificity of amplification. The detection threshold cycle for each reaction was normalized against the expressed level of the rice reference gene *ACT1* (GenBank ID: AK100267) [58]. The relative expression level of each gene was calculated using the 2^-(ΔΔCt)^ method with some modifications [59]: ΔΔCT = (C_T_, _Target_ − C_T_, _Actin_) − (C_CK_, _Target_ − C_CK_, _Actin_). C_T_, _Target_ and C_CK_, _Target_ indicate the ΔΔCT values of the target transcript from the treatment and control samples, respectively; C_T_, _Actin_ and C_CK_, _Actin_ indicate the ΔΔCT values of the reference gene *ACT1* from the treatment and control samples, respectively. The mean expression values were calculated using the three technical replicate measurements of each target gene. Three biological replicates were performed for the RT-qPCR experiment.

### Detection of differentially expressed transcripts between the heat-sensitive and the heat-tolerant rice

To identify the differentially expressed transcripts in the heat-sensitive and -tolerant lines responding to high night temperatures, we first identified the differentially expressed transcripts (DEGs) using Cufflinks (http://www.cbcb.umd.edu/software/cufflinks/). The unigene expression levels were normalized by the Fragments Per Kb per Million fragments (FPKM) values [32]. The threshold P-value ≤ 0.05 and FC log2 ≥ 1.0 were used to determine the significantly differentially expressed genes [35,36]. Second, the transcripts obtained by the above criteria were further analyzed for the Relative Fold Change (RFC) between the heat-sensitive vs. -tolerant lines with RFC ≥ 2.0. The RFC values were calculated using the following methods: if the transcript showed up- / down-regulation in both the heat-tolerant and -sensitive lines and the fold change in the heat-tolerant line was higher than in the heat-sensitive line, this indicated that the relative fold change in the heat-tolerant line was opposite to that in the heat-sensitive line, and the RFC was calculated using formula A. If the transcript showed up-/down-regulation in both the heat-tolerant and -sensitive lines and the fold change in the heat-tolerant line was lower than in the heat-sensitive line, this indicated that the relative fold change in up-/down-regulation in the heat-sensitive line was opposite to the heat-tolerant line, and the RFC was calculated using formula B. If the transcript showed up-/down-regulation in the heat-tolerant line but down-/up-regulation in heat-sensitive line, the relative up-/down-regulating fold change was similar between the heat-tolerant and -sensitive lines, and formula C was used to calculate RFC.

Formula A: RFC = (HT Log2 − HS Log2)/ HS Log2;

Formula B: RFC = (HS Log2 − HT Log2)/ HT Log2;

Formula C: RFC = (HS Log2 + HT Log2)/ 2.

HS Log2 and HT Log2 in the formulas indicate the logarithm of a 2-fold change in the heat-sensitive line and the heat-tolerant line, respectively.

### Availability of supporting data

The raw reads produced by Illumina-sequencing method in this publication have been deposited in NCBI’s Gene Expression Omnibus (GEO) dataset and are accessible through GEO Series accession number SRP050315 [http://www.ncbi.nlm.nih.gov/Traces/study/?acc=SRP050315].

## Additional files

Additional file 1:
**Influence on the grain plumpness of the heat-sensitive and heat-tolerant lines at high night temperatures.**
Additional file 2:
**Size distribution of the assembled transcripts.**
Additional file 3:
**The cluster results of the 2-fold change values for each differentially expressed transcript from the heat-sensitive and heat-tolerant lines.**
Additional file 4:
**RT-qPCR analysis of 12 transcripts selected from heat-tolerant and -sensitive rice lines under high night temperature stress.**
Additional file 5:
**The base sequences and FPKM values for the 35 differentially expressed transcripts.**
Additional file 6:
**The differentially expressed HTRTs without reference genes and/ or functional annotation.**
Additional file 7:
**The RT-qPCR primers for the 12 selected unigenes.**
