# Supplementary material for: Transcriptome changes in rice (Oryza sativa L.) in response to high night temperature stress at the early milky stage
Source: BMC Genomics. 2015 Jan 23;16(1):18. doi: 10.1186/s12864-015-1222-0 (PMC4369907; doi:10.1186/s12864-015-1222-0)

Additional file 1

Influence of high night temperatures on rice grain plumpness of heat-tolerant and -sensitive lines. Rice plants were exposed to high night temperatures for 20 h (two dark periods) at the early milky stage. Then they were moved to normal growth conditions and kept under such until maturity. Three biological replicates of temperature treatments were carried out. After maturation, thousand grains from each sample were randomly selected and used to calculate grain plumpness (GP).

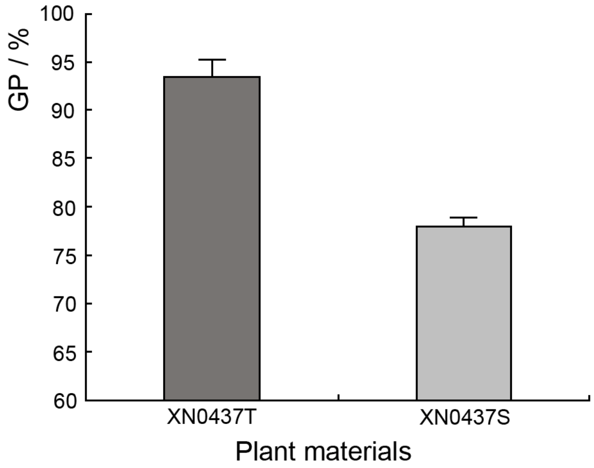

Supplement: Additional file 1: — Influence on the grain plumpness of the heat-sensitive and heat-tolerant lines at high night temperatures. [file 12864_2015_1222_MOESM1_ESM.pdf]
