# Supplementary figures and images for: Transcriptome changes in rice (Oryza sativa L.) in response to high night temperature stress at the early milky stage
Source: BMC Genomics. 2015 Jan 23;16(1):18. doi: 10.1186/s12864-015-1222-0 (PMC4369907; doi:10.1186/s12864-015-1222-0)

**Additional file 2**

**Size distribution of the assembled transcript size.**

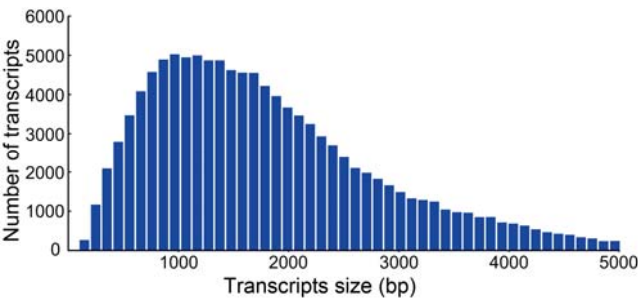

Supplement: Additional file 2: — Size distribution of the assembled transcripts. [file 12864_2015_1222_MOESM2_ESM.pdf]
