# Supplementary material for: Transcriptome changes in rice (Oryza sativa L.) in response to high night temperature stress at the early milky stage
Source: BMC Genomics. 2015 Jan 23;16(1):18. doi: 10.1186/s12864-015-1222-0 (PMC4369907; doi:10.1186/s12864-015-1222-0)
Supplement: Additional file 3: — The cluster results of the 2-fold change values for each differentially expressed transcript from the heat-sensitive and heat-tolerant lines. [file 12864_2015_1222_MOESM3_ESM.pdf]

Additional file 3

The cluster results of the 2-fold change values for each differentially expressed transcript from the heat-tolerant and -sensitive lines. The FPKM values and functional annotation of the differentially expressed transcripts from three biological replicates were performance for the cluster analysis.

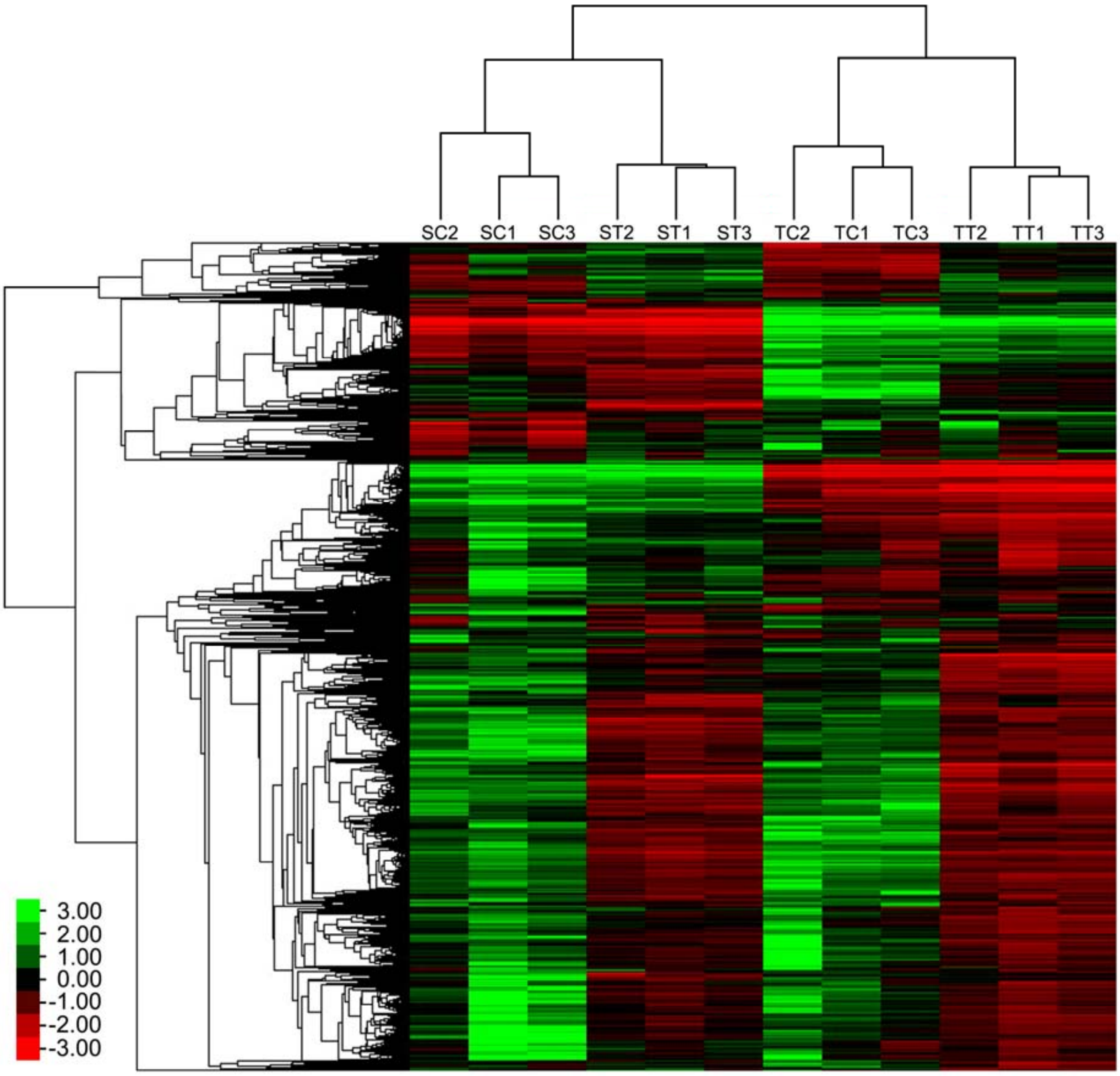

ST, SC, TT and TC indicate the treatment and control of the heat-sensitive and -tolerant lines, respectively; 1, 2 and 3 indicate three biological replicates.
