# Supplementary material for: Transcriptome changes in rice (Oryza sativa L.) in response to high night temperature stress at the early milky stage
Source: BMC Genomics. 2015 Jan 23;16(1):18. doi: 10.1186/s12864-015-1222-0 (PMC4369907; doi:10.1186/s12864-015-1222-0)

Additional file 4

RT-qPCR analysis of 12 transcripts selected from heat-tolerant and -sensitive rice lines under high night temperature stress. The magnitude of changes in gene expression as indicated by RNA-seq data analysis and RT-qPCR were compared using a column diagram. The x axis indicates plant materials; the y axis indicates fold change in gene expressed. XN0437S indicates the heat-sensitive line; XN0437T indicates the heat-tolerant line.

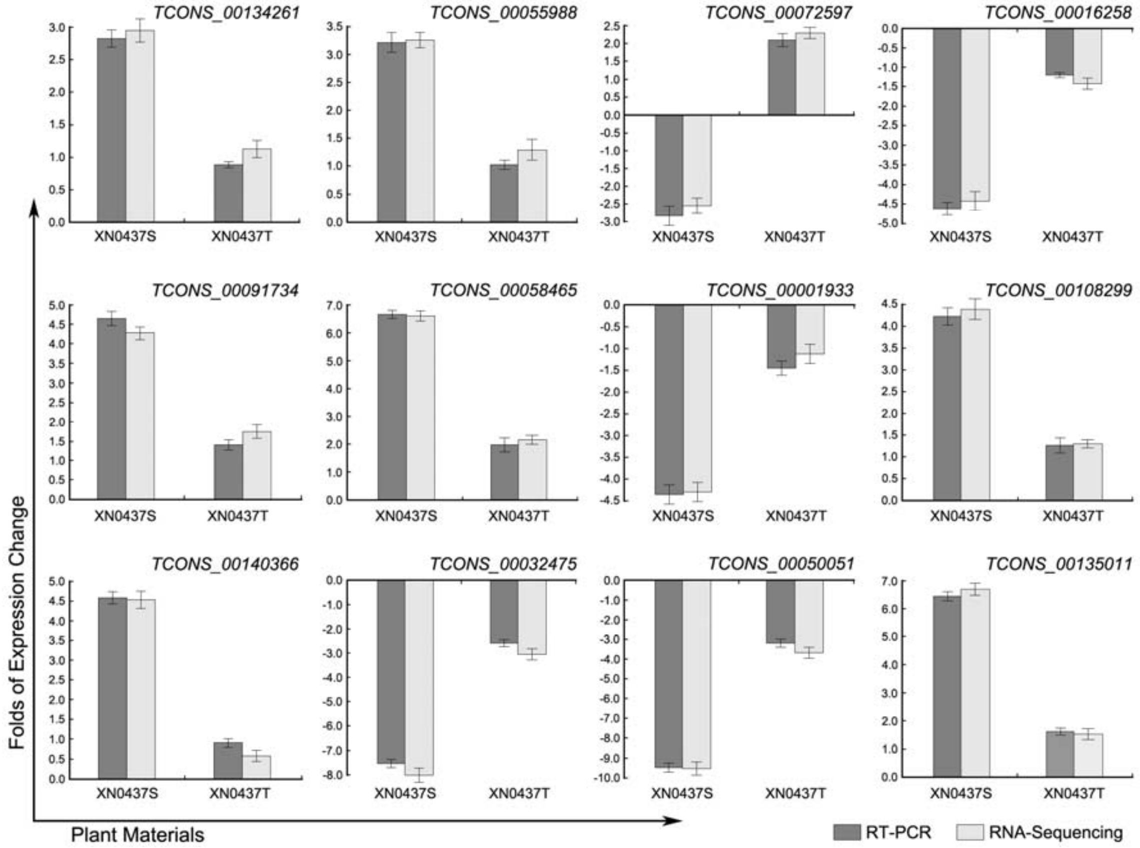

Supplement: Additional file 4: — RT-qPCR analysis of 12 transcripts selected from heat-tolerant and -sensitive rice lines under high night temperature stress. [file 12864_2015_1222_MOESM4_ESM.pdf]
