# Supplementary material for: Transcriptome changes in rice (Oryza sativa L.) in response to high night temperature stress at the early milky stage
Source: BMC Genomics. 2015 Jan 23;16(1):18. doi: 10.1186/s12864-015-1222-0 (PMC4369907; doi:10.1186/s12864-015-1222-0)
Supplement: Additional file 5: — The base sequences and FPKM values for the 35 differentially expressed transcripts. [file 12864_2015_1222_MOESM5_ESM.pdf]

Additional file 5

The base sequences and FPKM values for the 35 differentially expressed transcripts. "ST", "SC", "TT" and "TC" indicate the treatment and control of the heat-sensitive and -tolerant lines, respectively

TCONS_00050124; FPKM value: TC=1.95829, TT=16.4068, SC=5.49825, ST=1.04574.

GCATCTCCTCCATGGCACTCACCTCACCACACACCACTCCACCATCACTCACAGCCACCACCCGCCGCCATGGAGCAGGTCTCGTGCTTCGCCGCCGCCG
CCGCCGCCGTCCTCGTTGTTCTCTCGCTCGCTCGCATGCTCCTCGCGCCGCGCAGGGAGTGGGACGGGCTGAACCTGCCGCCCAGCCCGTCGCGGCTGC
CGTTCATCGGCAGCTTCCACCTCCTCCGCCGCAGCCCGCTCGTCCACCGCGCGCTGGCCGACGTCGTGCGGCAGCTCGGCGCGCCGCCGCTCATGTACAT
GGAGATCGGTGAGGTCCCGGCCATCGTCGTGTCGTGCGCCGACGCGGCGCGCGAGATCATGAAGACGCACGACATCAACTTCGCGTCGCGGCCGTGGCC
GCCGACTGTCCAGAAGCTGCGCGCGCAGGGGAAGGGCATCTTCTTCGAGCCCTACGGCGCGCTGTGGCGCCAGCTCCGCAAGATCTGCATCGTCAAGCT
GCTCAGCGTCCGCCGCGTCAGCTCGTTCCACGGCGTCCGCGAGGAGGAGGCCGGCCGCCTCGTCGCCGCCGTCGCCGCGACGCCGCCGGGACAGGCCG
TGAACCTCACCGAGCGGATCAAGGTGGCCATCGCGGACACCACGATGCGCCCCATGATCGGTGAGAGGTTCGAGAGGCGGGAGGACTTCCTCGAGGTC
CTCCCGGAGATCGTCAAGCTCGCCTCCGGGTTCAGCCTCGACGACCTGTTCCCGTCGTCGTGGCTCGCCGGCGCCATCGGCGGCTCGCGGCGAGGCGGT
CAACCGGGCGTCCTACGAGCTGGTGGACAGCGCCTTCCGCCAGCGGCAGCAGCAAAAGGAGGCCATGGCCGCGCCGCCGCCTGACATCGCCAAGGAGG
AGGAGGATGACTTGATGGATGAGCTCATCAGGATACACAAGGAAGGTAGCCTCGAGGTGCCTCTTACTGCCGGCAACTTGAAAGCTGTCATCCTAGAGCT
CTTTTGTGCGGGAAGTGAGACATCCTCGAATGCGATCCAATGGGCAATGTCAGAGCTGGTGAGGAACCCAAAAGTGATGGAGAAAGCACAGAACGAGG
TGCGAAGCATCCTCAAAGGGAAGCCCACAGTGACTGAGGCCGACATGGTAGACCTAACGTATGTGAAGATGATTGTCAAGGAGACTCATAGGCTGCATC
CGGTATTGCCATTGCTCACCCCAAGGGTGTGCCAGCAAACTTGCCAGATCATGGGGTATGACGTGCCCCAAGGCAGTGTCATATTCATAAAGTCGTGGGC
AATAATGAGGGACCCCAAACATTGGGATGACGCCGAGACATTCAAACCAGAGCGGTTTGAGGATAGTGAGATCGATCTCAAGGGCACAAACTATGAGTT
CACTCCATATGGGGCCGGACGCAGGATCTGCCCTGGCTTGGCATTAGCCCAGGTCAGCATAGAGTTCATCCTCACCATGCTGCTCTACCACTTTAACTGGG
AACTCCCTAACGGGGCAGCACCGGAGGAGTTGGACATGACTGAGGACATGGGCCTCACCATCCGAAGGAAGAATGATCTCTACCTGCTCCCAACTCTTC
GTGTGCCTCTTACCGCATAGCACCTACCTTCCTCAATGTGCGCAATCAGTATCTATGCTTGTGATGCCTCTACATAAGATCTTACAAAATGTACCTCGCCACT
CGTGGCTGCCTTAATAAACTCTCCTTGTTTTTCAATGTTTGATTTTCTTAATAAGGTTATGTGTTTCTTTGTTGTGAT>

TCONS_00134261; FPKM value: TC=3.56559, TT=6.58514, SC=0.764641, ST=5.60841.

AAGCTAGCAACCAAGCAAAATAGGAGCTAGTAAGCAAAGCAAGAACTAGATCGATCTCTGACAGGGAATGGCCATGGGATTGTTAGCGTGGATGGTGGC
AGCGGCCGCGGCGGCGGTGCTGGCGTCGTGGGCGTTCAGCGCGGTGGTGCACCTGGTGTGGAGGCCCCGCGCCATCAGCAGGCGCCTCCGGGCACAGG
GCGTGGGCGGGCCGGGCTACAGGTTCTTCTCCGGCAACCTCGGAGAGATCAAGAGGTTCCGCGGCGATGGCGCTGGCGTCGTGCTCAACGTCTCCTCCC
ATGACTTCCTCCCCATTGTACAGCCACATTTCCGCAAGTGGATTCCCCTATATGGAAGGACATTCCTGTATTGGTTCGGAGCACAGCCAAACATATGCTTGG
CTGACGTGAGCATGGTGTGGCAGGTATTGTCAGACCGGACGGGGATATACCCCAAGAACCTGACGAACCCTCACTTTGTGCGTCTACTTGGCAAGGGGC
TTGTGCTCACTGACGGCGATGAGTGGAAGCGCCACCGCAAGGTGGTCCACCCGGCCTTTAACATGGACAAGCTCAAGATGATGACGATGACCATGTCTG
ACTGCTCTCGGTCCATGATGTCGGAGTGGGAATCGGAGTTGGCGGCGAAGGGTGGTCTTGTTGAGATTGAGCTAAGCAGGCGGTTCGAGGAGCTCACTG
CCGATGTGATCTCGCACACGGCGTTTGGTAGCAGCTACAAGGAGGGGAAGCAGGTCTTCCTGGCACAGAGGGAGCTCCAATTTCTTGCCTTCTCCACCTT
CCTCACTGTTCAAATTCCAGGGTTTAGCTACCTTCCGACCATGAAAAACTTCAAGACATGGTCGCTCGACAAGAAGGTGAGGGGCATGCTCATGGACATC
ATCAAGACCCGGCATGCCAACAAGGATGTAGCTGGGTATGGGAATGACCTACTTGGGTTGATGCTGGAGGCATGTGCGCCGGAGCACGGGGAGAGCTGC
CCACAACTGAGCATGGATGAGATCATTGACGAGTGCAAGACCTTCTTTTTTGCAGGGCATGACACCACATCACACTTGCTCACCTGGACCATGTTCCTGC
TGAGCACGCACCCAGATTGGCAGGAGAAATTAAGGGAGGAGATTGCAATGGAGTGTGGTGACAAGGTGCCCACTGGTGACATGCTCAACAAGCTAAAG
ATGGTCAACATGTTCCTCCTAGAGACCCTAAGGCTGTACAGCCCTGTCTCACTCATACGGAGGAAGGTTGACACTGATATTGAGCTCGGCGGCATCAAGA
TGCCTGAGGGTGCACTATTGACGATCCCGATCGCGACAATTCACCGTGACAAGGAAGTGTGGGGGGAGGATGCGGATGAGTTTAGGCCGGAGAGGTTCG
AGAATGGGGTGACGAGGGCAGCGAAGCACCCCAACGCACTACTCTCCTTCTCCAGCGGACCGAGGTCATGCATTGGGCAGAACTTTGCAATGATCGAGG
CCAAGGCTGTCATTGCCATGATCCTACAGAGGTTCTCCTTCACTTTGTCCCCCAAGTACGTCCATGCACCCACTGATGTGATCACGCTGCGTCCCAAGTAC
GGGCTCCCTATGATCCTCAAGAGCCTCAAGCTGTAGGGAGAAATACGTAAGAAATATATGAATTGTCCTTTCTCTTCTATAAGAGTTTGCCACTTCGCTGGT
AGGAGTTATTTAGAACTAATTGGTTGTACATGATACATCAATTTTGTTGATGTTATTCAGACAATGTATTTGTATATTTCATTTTAACAAAATTTGGATGTGGA
TTTGCTTTTTG>

TCONS_00070653; FPKM value: TC=6.57927, TT=12.316, SC=4.57245, ST=31.8206.

CGCCAACGAGGCAACGAGACCCGTAATGCAACGATCGCATCTGCGTTTCAGGCGTCAGCCATGGCGTCTGCAGAGATGCCTGGATTGTCTCCGCAAGATC
TGATCCATTTCATCTCCTTCTAGAAGCACAAGCGCCGCTCGGTATAAAGGCAGACGCATTGTCACAAATAGCTGCAGTGCACCAGAGTCACAGAAACACA
TCACACATTCGTGAGCTCAGCTTAGCCATGGATAACGCCTACATTATTGCCATTCTCTCTGTAGCTATCCTCTTCTTGCTCCACTACTACCTCCTCGGCCGCG
GCAATGGCGGGGCGGCGCGGCTGCCGCCGGGTCCACCGGCCGTCCCGATCCTGGGACACCTCCACCTCGTCAAGAAGCCGATGCACGCCACCATGTCCC
GCCTCGCCGAGCGGTACGGGCCGGTGTTCTCGCTGCGCCTCGGGTCGCGGCGCGCCGTGGTGGTGTCGTCGCCGGGGTGCGCCAGGGAGTGCTTCACCG
AGCACGACGTGACCTTCGCGAACCGGCCCAGGTTCGAGTCGCAGCTGCTGGTCTCGTTCAACGGCGCCGCGCTCGCCACGGCGAGCTACGGCGCGCAC
TGGCGCAACCTCCGCCGGATCGTCGCCGTGCAGCTGCTCTCCGCGCACCGCGTCGGCCTCATGTCGGGGCTCATCGCCGGCGAGGTCCGCGCCATGGTG

CGGAGGATGTACCGCGCCGCGGCCGCGTCCCCCGCCGGCGCCGCGCGCATCCAGCTGAAGCGGAGGCTGTTCGAGGTCTCCCTCAGCGTGCTCATGGAG
ACCATCGCCCACACCAAGGCGACCCGCCCCGAGACGGACCCGGACACCGACATGTCCGTGGAAGCCCAGGAGTTTAAGCAGGTCGTCGACGAGATCAT
CCCGCACATCGGCGCGGCCAACCTGTGGGACTACTTGCCGGCGCTCCGGTGGTTCGACGTGTTCGGCGTCAGGAGGAAGATCCTCGCCGCTGTAAGCCG
GAGGGACGCGTTCCTTCGCCGCCTGATCGACGCGGAGCGGCGGAGGCTGGACGACGGCGACGAGGGCGAGAAGAAGAGCATGATCGCCGTGCTGCTC
ACTCTGCAGAAGACAGAGCCGGAGGTGTACACCGATAACATGATCACAGCTCTAACGGCGAACTTGTTCGGAGCAGGAACAGAGACAACCTCGACGAC
ATCAGAATGGGCGATGTCGCTACTGCTGAACCACCCCGACACACTCAAGAAAGCGCAAGCCGAGATCGACGCATCCGTCGGCAACTCTCGCCTGATCAC
CGCCGACGACGTGACTCGCCTCGGCTACCTCCAGTGCATCGTCAGGGAGACGCTCCGCCTGTACCCCGCCGCGCCGATGCTCCTCCCGCACGAGTCCTCC
GCCGACTGCAAGGTCGGCGGCTACAACATCCCGCGCGGGTCGATGTTGCTCATCAACGCGTACGCCATCCACCGTGACCCGGCGGTGTGGGAGGAGCCG
GAGAAGTTCATGCCGGAGAGGTTCGAGGACGGCGGGTGCGACGGCAATCTCTTGATGCCGTTCGGGATGGGGAGGCGGAGGTGCCCCGGCGAGACGCT
GGCGCTGCGCACAGTGGGGTTGGTGCTGGGCACGCTGATCCAGTGCTTCGACTGGGAGAGGGTCGACGGCGTGGAGGTCGACATGACTGAAGGTGGCG
GGCTCACCATCCCCAAGGTCGTGCCGTTGGAGGCCATGTGCAGGCCGCGCGACGCCATGGGTGGTGTTCTTCGCGAGCTCGTCTGAATATTTTTTGGCGG
CGTTTGCATCTCCAGGACGAACTCATGTATTGAAAGCACCAAAAGTAAGTAGCAAATAAGCTTCTCGTGAGCATACACATAACACATGTGAGCTTGTAATG
TGGAATAAATTACACGTAGAGGATTTGGAAGAGAGTGACTGCGCTAGCAATCGCTCTTTGAGAGTTGTGTTTTACAGTTTTAGTGAGGA>

TCONS_00055988; FPKM value: TC=2.45668, TT=5.10281, SC=0.229333, ST=2.39404.

CTGAGCTTCTAGCTCGCGAGTGATCTCTTCTTCTAGCTCAGTAACCACAGCTACAACCTACAGCTTACAGTGTCCGCAAAATTGTAACATTGTGTCCTTCC
ATCGGCAACAATGGCGGCCTCGGTGGAGTACAAGCTGGCTCCGCACCCGTGGGCGAGCAATGCTCCGTCGAGCAACCTCGACTTGTTCCCGTCCGGCGG
CGGCAAGCGCCGTTCGGGCTCCGAGACCGACTCCGACGACGAGGACAGCATACCACCGGACTGGAGGTCGCTGTACCACCCGCGGCTGGAGGTGGCGG
AGCCGGCCGTCAAGGACCCCCGCGACGAGGCCACCTCCGACGCGTGGGTGCGTCGCCACCCGGCGCTCGTCCGGCTCACCGGCAAGCACCCGTTCAAC
TCCGAGCCGCCGCTGCCGCGCCTCATGTCGCACGGCTTCATCACGCCGGCGCCGCTCCACTACGTGCGCAACCACGGCGCCGTGCCCAAGGCGGACTGG
TCGACGTGGGCCGTGGAGGTGACCGGGCTCGTCAAGCGCCCCGCGAGGCTCAACATGGAGCAGCTCGTGACCGGGTTCGAGGCCGTGGAGCTCCCCGT
CACGCTGGTGTGCGCGGGCAACCGGCGCAAGGAGCAGAACATGGTGCGCCAGACCGTGGGCTTCAACTGGGGCCCCGGCGCCATCTCCACCTCCGTGT
GGCGCGGCGTGCGGCTGCGCGACGTGCTGCGGTGGTGCGGTGTCATGGGCGCCTCCGCCGGCGCGGCCAACGTGTGCTTCGAGGGCGCCGAGGACCTC
CCAGGCGGCGGCGGGTGCAAGTACGGCACCAGCCTGCGCCGCGAGGTGGCCATGGACCCCGCCCACGACGTCATCCTCGCCTACATGCAGAACGGCGA
GCCGCTCACGCCCGACCACGGCTTCCCCGTCCGGGTCATCGTCCCGGGCTTCATCGGCGGCCGCATGGTGAAATGGCTCAAGCGCATCATCGTCGCGTCC
AGCGAGTCGGAGAGCTACTACCATTACCGCGACAACCGCGTCCTCCCGTCTCACGTCGACGCCGAGCTCGCCAATGCCGAAGCTTGGTGGTACAAGCCG
GAGTACATGATAAACGAGCTGAACATAAACTCGGTGATCACCACGCCGGGACACGATGAGGTGCTCCCCATCAATGCGCTGACGACGCAGCGGCCGTAT
ACGATGAAGGGATACGCCTACTCCGGCGGTGGCCGGAAAGTTACAAGGGTAGAGGTGACCCTGGACGGCGGCGAGACGTGGCAGGTGTGCAACCTTGA
CCACCCGGAGAGGCCGACCAAGTACGGAAAGTACTGGTGCTGGTGCTTCTGGTCCGTCGATGTCGAGGTGCTCGAGCTGCTCGCCGCCAAGGAGATCGC
CGTCCGCGCCTGGGACGAGTCCCTCAACACCCAGCCGGAGAAGCTCATTTGGAATCTCATGGGCATGATGAACAACTGCTGGTTCAGGGTGAAGACGAA
GACGTGCAGGCCGCACAAGGGGGAGATCGGGCTGGTGTTCGAGCACCCGACGCAGCCGGGCAACCAGGCCGGCGGGTGGATGGCGAGGCAGAAGCAC
CTCGAGACGTCGGAGAGCGCGGTGAGCACGCTGAAGCGCAGCACGTCCACGCCGTTCCTCAACACGGCCACCACGCAGTACACCATGTCCGAGGTGCG
CCGCCACACGACGCCGGAGTCCGCCTGGATCATCGTGCACGGCCATGTCTACGACTGCACGGGGTTCCTCAAGGACCACCCCGGCGGCGCCGACAGCAT
CATGATCAACGCCGGCACCGACTGCACCGAGGAATTCGACGCCATCCACTCCGACAAGGCCCGTGGCCTCCTCGAGATGTACCGCATCGGCGAGCTCAT
CGTCACCGGCAGCGACTACTCGCCGCAGAGCAGCAGTGCTGACCTCACGTCCATCGTTGAGAGCCCTACGGCAGCGGCGGCGCCCGCCGTGCCGGTGTC
GACCGTCGCGCTGTCCAACCCGCGGGAGAAGGTGAAATGCCGGCTCATGGACAAGAAGAGCCTGTCCTACAACGTGCGCCTGTTCCGGTTCGCGCTGCC
GTCGCCGGACCAGAAGCTCGGGCTACCGGTCGGCAAGCACGTGTACGTGTGCGCGTCGATCGGCGGCAAGCTCTGCATGCGCGCGTACACGCCGACGA
GCTCCGTCGACGAGGTCGGCTACATCGAGCTCCTGATCAAGATATACTTCAAGGGCGAGGACCCCAAGTTCCCCGACGGCGGGCTCATGTCGCAGTACCT
GGACTACCTGCCGCTCGGCGCCACCATCGACATCAAGGGCCCGATCGGGCACATCGAGTACGCCGGCCGCGGCGCCTTCACGGTGAACGGCGAGCGCCG
GTTCGCGCGGCGGCTCGCCATGGTGGCCGGCGGGACGGGGATCACGCCGGTGTACCAGGTGATCCAGGCCGTGCTCTGGGACCAGCCCGACGACGGAA
CGGAGATGCACGTGGTGTACGCGAACCGGACGGAGGACGACATGCTCCTCCGGGAGGAGATCGACCGGTGGGCGGCGGCGCACCCGGCGCGGCTCAA
GGTGTGGTACGTGGTGAGCAAGGTGGCGCGGCCGGAGGACGGGTGGGAGTACGGCGTGGGGAGGGTGGACGAGCGGACGCTCAGGGAGCACCTGCCG
CCGGGCGACGGCGAGACGCTCGCGCTCGTGTGCGGGCCGCCGGCGATGGTCGAGTGCACGGTGCGGCCGGGCCTGGAGAAGATGGGCTATGACCTCGA
CAAGTCCTGCCTCGTCTTCTGAAACGTGACGTGTGTAGTGTACGACACTGCAAAAACTGTACATATACGTAGCATAGTTGCATAAATTTTAATTCAAAGTT
TGGAGCTCTCTCGAGTTCCGGTACATCGGAGTAGCGCGCGGCGGTCGCATTCGATGGCCATGGAAAAAGTGTAGAGTGTAGATTGATGGATGGTTGTGCA
CATGGTTCATTTGAGAATCAAGATGTATATTTCATTGGTCATACTAGAGCAAATAAAACCGAGATATTACTACGACTGTAACAATCTGTTATTACATTGTGTT
GACTGTCGGCAAAACTCACTCCTCTGCCTGATATTTTTCAAATGTTTGGAAATGGCTAATGTTTTCTTCTTGCATCACTAACCAAATTGTTGAATTGAAGTG
AGGCCCATATAGAGGTCATCAGAATAGGCCCACTGGCAGCCCACAAGATAATTTACCAGCCCATTAATCAAATCTTCC>

TCONS_00100761; FPKM value: TC=2.70725, TT=5.14761, SC=1.2985, ST=9.39605.

CAACCATCTCGTCAACAAAAAGGCGCCTCTAAGAATTCCTCCCAAACTTTTTGGTGTTGTCAAGCCGTGCGTTTCGGGGTACATCAACGGCGGCGATGAG
CTCCCGGATGGCCGGATCGGCGATCCTCCGTCACGTCGGCGGCGTCCGCCTCTTCACCGCGTCGGCGACCTCTCCGGCGGCGGCGGCGGCGGCGGCGGC
GAGGCCGTTCCTTGCAGGCGGAGAAGCCGTCCCCGGGGTGTGGGGGTTGCGGCTGATGTCCACGTCGTCCGTGGCGTCGACGGAGGCGGCGGCCAAGG
CGGAGGCCAAGAAGGCGGACGCGGAGAAGGAGGTGGTGGTCAACAGCTACTGGGGCATCGAGCAGTCGAAGAAGCTGGTGCGGGAGGACGGCACGG
AGTGGAAGTGGTCTTGCTTTAGGCCATGGGAGACCTACACCGCGGACACTTCGATCGATCTGACGAAGCACCACGTGCCCAAGACGCTGCTCGACAAGA
TCGCCTACTGGACCGTCAAGTCGCTGCGCTTCCCCACTGATATCTTCTTCCAGAGGAGGTATGGCTGCCGCGCGATGATGCTGGAGACGGTGGCGGCGGT
GCCGGGGATGGTGGGCGGCATGCTGCTCCACCTCCGGTCCCTCCGGCGCTTCGAGCAGAGCGGCGGCTGGATCCGCACGCTGCTGGAAGAGGCCGAGA

ACGAGCGCATGCACCTGATGACCTTCATGGAGGTGGCGAACCCAAAGTGGTACGAGCGCGCCCTCGTCATCACCGTCCAGGGCGTCTTCTTCAACGCCT
ACTTCCTGGGTTACCTCCTCTCCCCCAAGTTCGCGCACCGCGTCGTCGGCTACCTCGAGGAGGAGGCCATCCACTCGTACACCGAGTTCCTCAAGGACCT
CGAGGCCGGCAAGATCGACAACGTCCCTGCCCCGGCCATCGCCATCGACTACTGGCGCCTCCCCGCCAACGCCACGCTCAAGGACGTCGTCACCGTCGT
GCGCGCCGACGAGGCTCACCACCGCGACGTCAATCACTTCGCATCGGACATCCATTACCAGGGCATGGAGCTGAAGCAGACCCCTGCGCCGATCGGATAT
CACTGAGGATGTTTGTCTACTGCCGAGGATTTGCATCACTAATAATCAAATCAAATGAGTATACTTCGTTTTTCAGGGAAAGCAAATGAGAGCTCTTTAGC
TGTATCGGATTTGGTAATTGCTACTAGTAACTACTAAGTACTGAAAAATAAGGAGTCTCCTTGTTTGAAGTACAAATCTAGGAGATTTTTTGAACCAACTCT
ATCTGAATTGGGCCTGTTTTTCGTACGCCGCACTGCTTCATGTACATATATTCCATCCCCTATCTTATGTGTGGCAAGTGTCTTTCTAAATTCTTGGATGTAAT
GACACCTGAATTTCTTTCGTTGAACTATTGGCAGCTGAGTTATATATACTACATACCATTCTTTTGTTAAAGCAGATGCTGGATGCTATCTTGTTTGTCTTCTT
CCAAATTGCCTGTTGTGACGCGGACGGCGTACAGAGATGGAGTCGATCGATCTCACCGGTCCCAATCCGCCGCCTTCTCCTTCGCCGACATGTACACCCT
ACACCTGCAGCGTCCCGCGGTCACCCTCTACCCGCTCGGAAACCCAGCGCCGCCGCCGCCGCGCCGCTGGTGCCGCTCACCACTGTGCCCGCTCCCCGT
GAGCGCGGCCACCGCAGGGAGCAGGCTCGCTTATATCCCCCGCGCCTCCGCCGCCGCAAACCCGCATCCCGACCCTCAGGATGAGCCGGAGCCGCAGCC
GAAGGGCGGGTTCTGGACCAGGTGGATGGCGGAGAGCGCGGAGATGAGGGCCAAGGTGGCCAAGCTCGGCCTGGCCGCCGTGCTGGCCTACGGGCTGT
TCGACGCCGTCACCTACACGACGTTCTTCGTGCTGGCCTTCCTCGGCTACGAGAAGAGCACCGGCAAGAACCCGGCGGCCAATCTCAAGGCCTTGCTGG
GGATAGTTATCCTAATGTGGACCGGTAACAATGTTACTAGGCCATTCCGAGTTGCTGGTGCAGCTGCTTTGGCACCGGCGATCGACAAAGGGTTGAAGGG
TATTCAAGAGAAACTTAACCTTCCAAGCCAGATGTATGCTTTTGCACTCGTGGTTGGCTCCGTGGCTGCGGTATGCTTCACAATTTTTGGATGCTTGATCCT
CTCGAAATGGGGAAAGTGATTTGTTGGAATCCTGGCCATCAAGCATGATTGGTATGAATATTGGATCCAACTAGGTTAGTCTAGTTTATTAGAATCTTCTG
TTTCCCATGCTCTTTAAGATGATGTTAGTTTTCTAAACATTATTCAGTTGGTTAATTGACAAGATATAAAAGTTACTTGTCCCATTTATGCAGTTGGGTAACA
ATAGTTTTGTTTTGTTTCTAGTTAGTTTTGCGAAAG>

TCONS_00072597; FPKM value: TC=11.9037, TT=1.59872, SC=2.27429, ST=9.83814.

GCAAAGTATAGCAGCAAACAAAGCCAGCTTGTCACTGCGCTTCTTTCCTAGTTTCCTTCTCAATTCTCATTCTCAAAAGAGAATATATTGAGCTGATCGAG
CTAGATATATAGAAGCACTAGCTAGTTGATCAACAATGGAGCACAGCTTCAAAACCATAGCAGCTGGAGTGGTGATCGTCGTGCTGCTCCTGCAGCAGGC
GCCCGTGCTGATTCGGGCCACCGACGCGGACCCTCTCCAGGACTTCTGCGTCGCCGACCTCGACAGCAAGGTCACGGTGAACGGGCACGCGTGCAAGC
CGGCGTCGGCCGCCGGCGACGAGTTCCTCTTCTCCTCCAAGATTGCCACGGGCGGCGACGTGAACGCCAACCCGAACGGCTCCAACGTCACGGAGCTC
GACGTCGCCGAGTGGCCCGGCGTCAACACGCTCGGCGTGTCCATGAACCGCGTCGACTTCGCGCCCGGTGGCACCAACCCGCCGCACGTCCACCCGCG
CGCCACCGAGGTCGGCATCGTGCTCCGCGGCGAGCTCCTCGTCGGCATCATCGGCACCCTCGACACCGGGAACAGGTACTACTCCAAGGTGGTCCGTGC
CGGCGAGACGTTCGTCATCCCGAGGGGGCTCATGCACTTCCAGTTCAACGTTGGCAAGACGGAGGCCACCATGGTGGTGTCCTTCAACAGCCAGAACCC
CGGCATCGTCTTCGTCCCGCTCACATTGTTCGGCTCCAACCCGCCCATCCCGACGCCGGTGCTTGTCAAGGCACTCCGCGTGGATGCTGGTGTAGTTGAG
CTGCTCAAGTCCAAATTCACCGGCGGGTACTAATTAATCCGAGAGCATTTTCATGGTGTTCTCTGCTACCAGCAGCATACTTTAAGCTCCCGTTGTGTGGTT
GCATGTTATTGGATTGAATAAACTTTCTCCCATGTTATGGGAATGGAAATAAAATGTAATACTTCTTTGTGATGTTTTTTTTCTCCTTATAATGAATTAAGAAT
CGTGTATCTTTTCTGTGTATGTGCAATTATCATCATGTTGTTTTGAAATTGTATTTTCAGTATTTATATATCCTATCTTACTATAAAGCTGATGTCC>

TCONS_00016258; FPKM value: TC=3.29762, TT=0.131122, SC=3.48879, ST=1.44299.

CAAGGATCAAATAGCCAAATGGATTTTGTCTTTTCAAGTACATCCTAAGACAGACAATGAATTAGATAATGGACAATTTTATGGATTCTGTGGCTCTAGAAC
AACTCAGTTTCCCTCAACTAACATGAAGGACCCTTGCCACAATGGTAGTCATCTTGCAAGCACTTATTCTGCCCTTGCTATCCTGAAGATTGTAGGTTATGA
TTTAGCAAACATTGATAACAAGGTTCTTTTGTCCTCAATGAGAAACCTCCAACAGCCAGATGGAAGCTTCATGCCTACTCATATTGGTGCGGAAACAGAC
CTACGCTTTGTATACTGTGCAGCTGCAATCTGCTCGATGCTAAAAGATTGGACAGGAATGGACAAGGAAAAGGCCAAGCAATACATTCTTAGTTGCCAGT
CATACGATGGTGGCTTTGGCTTGGTTCCTGGTTCAGAATCTCATGGTGGAGGGACATTCTGTGCTGTTGCAGCTTTGTGCCTGATGGGTTTCATTCAAGTT
GATTTGGCATCAAATTTACAAGAACCATCATCAATTGATGTACGCTTGCTCCTGGAGTGGTGTCTTCAGAGACAAGCGGCAGACGGAGGTTTTCAAGGAA
GAAGGAACAAATCCAGTGACACATGCTATGCATTTTGGATTGGAGGTGTCCTAAAGATCATCGGTGCATACCGCTTCATTGATCATGGTGCTTTGCGAAGT
TTTTTGCTCTATTGCCAATCACCTTATGGAGGTTTTACAAAGTTTTTGTACGATCAATTCCCGGATATCTACCATTCTTACTATGGACTTGCCGCTCTTTCCTT
GCTGGAAGAGGAAGGGCTGGAGCCACTCTGCACCGAACTGGGGATCCTCTCTGCTGCATTGTAGCTTGGAATTGATTGTTCATCTTCTGTATTCAGTCAA
GTTATGGTGTTCTTTAAAGTCCATTCTTGTAATCTAATACAACATACAGCTGAAAAGAATGCAAGTAATCTATAGAGTTCGTTTGTTTGTTTGGGTAGGCAG
GTCATGCAACATTGTCTTGGATAATATGATTGTGAAGTACAGAGGAGCATTCTGTAAAGGCTATTTCATTTTCAGTTAGTCGAACTAACTGATATTCTGATAG
ACAGTTGAGTTGGCTGAGCAGTTCTATCACTTGGAAAGCTATTGAAAACAACTCATGTAACAGCTGAACAAAATTATCATCTTTATTCTGTTTCTTGATGTG
CTTCCTATGTATCACTGATTAATTCTGGTAGTTGTAGATGCTGATATACACACACAGACACACAATTTCTTTTCTTTTGACAGGCCATCAGGTTAACAGCATA
ATACTTGTGATTGAGAACAGAGAGAGAAATGAGAGATTTTTTTTTACTGAACCTGCGTGTCTGAACTCTTCTGAAGATAAAATTGAAAGTTGCAGTACTG
AACTGAATTTGCAGCAGATCACATGGATGATATTGGCGGGAGAGCCGGCTCAGTCAGCTGCAGCTGCCATAACTGCCACCGCCACCCCCACATCCACTCC
GATTCATTGCGAATTCAGTTTGATCAGTGGTTGGTCGGTTGGCCAGCCTCCCATCCACCGGAATTCTCAAATCGGAGCAAGCAGCCTACTGATTGCGATTG
CATCCTCTGATCTGATCTGCTGATGCGAATGCGAGCAGTAATCAACAGCGTAGTAGATTCAGTCCACGCGCCCATTAAAGCCAAGCCACTGTCCTTGTCCA
TCTTTGTCCAGGCCTCGCGTTCCTCGGTTCCTCCTCCTCCTTGCTAGCTGCTGCTGAGTGCTCGGCGATGACGGGCGTCGGGTCGGCGGTGCGGCGGCTC
TACCTCTCCGTCTACAACTGGGCCGTCTTCTTCGGATGGGCGCAGGTGCTGTACTACGCGGTCACGACGCTGCTGGAGAGCGGCCATGAGGCCGTCTACG
CCGCCGTCGAGCGGCCGCTGCAGTTCGCGCAGACCGCCGCCTTCTTGGAGATTCTTCATGGGCTTGTAGGTTTGGTGAGGTCTCCAGTCTCTGCAACTCT
TCCACAAATTGGATCAAGGTTGTTTCTTACCTGGGGCATCCTGTGGAGCTTTCCAGAGACACATTCACATATCCTTGTTACTTCCTTGGTCATAAGCTGGTC
CATCACTGAGATCATTAGATATTCCTTCTTTGGCATGAAGGAGACATTCGGATTTGCCCCTTCCTGGCTCTTATGGCTGAGGTATAGCACCTTTATGGTATTG
TATCCTACTGGTATCAGCAGTGAGGTCGGTTTAATCTACATTGCCTTGCCTTACATGAAGGCAACAGAGAAATATTGCCTTAGGATGCCCAACAAATGGAA
CTTCTCCTTTGATTTCTCCTATGCATCCATTCTTTCTCTCGCCGTCTACGTGCCCGGATCGCCTCACATGTTCACCTACATGCTTGCCCAACGGAAGAAGGC

ATTGGCAAAGGCTAAGGCTGCATAATGGTGATGCTCAAGAGCTTTCTTGAATTTGTCACTTGTGGCTTAAGGATCTGCCTAAATAGCTCACATTTGCTACTC
TTTTAGTAGAAGTCTGTAGATGAGGACATGTTGGAAAGAACTACTATTACACTGCCCATCATTTGTTGTAACTGTAGTCTTCTACCAAGAATTCT>

TCONS_00119170; FPKM value: TC=5.56681, TT=9.88232, SC=2.50019, ST=14.445.

CGACGCTGATCAATCACAACCCCCTCTCCCCCTCCAAATCCTATTAATACCCACCAAATCCCCTCCAAATCCCCCCATCTCCTCCTCCTCCTCCTCCTAAGC
TCAAGCAAAGATTCATCCATCGATCCAACTGATCCAATCCAAACTCCAAAGGCAAAGCAAAGAAGGTCGCATTCGATTCGATTCCAGCGATGGCGGCGGT
GATGGTGGCGAGGCAGGGGAGGGAGCTGCAGAGGTACAGCGACAACACGGGGGGGAGGATGGTGGTGGGGTGCATCCCGTACAGGGTGAGGGGCGAC
GGCGGCGGGGTGGAGGTGCTCGTGATCAGCTCGCAGAAGAAGGGCGCCGCCGCCGGCGACGTGGTGATGTTCCCCAAGGGCGGGTGGGAGCTGGACG
AGTCCGTGGACGAGGCGGCGCGGCGGGAGGCCCTGGAGGAGGCCGGGGTGCTCGGCGAGATCGGCGCCTCGCTGGGGCGGTGGTGCTACCGGAGCCG
CCGCTACGACGCCACCTACGAGGGCTTCGTCTTCCCGCTCCGGGTCACCGACGAGCTCGACCGGTGGCCGGAGATGGCGGCCAGGCGCCGCAGCTGGG
TCTCCCCGCAGCAGGCCATGGACCGGTGCCCGCACTGGTGGATGCGCGAGGCCCTCCAGCGATTCGCCGACCTCTTCCCCCAACCCACGCCGTTGTCCCT
CTTGTAGGCGGCGGCGCCGGCGGCGAGTTCGCCGGAGTTGAGAGCGATGTAATTAATTTGCAACCTCACGGGTCACAAGAGATTCGGCCATGTAAATCTC
ACC>

TCONS_00078028; FPKM value: TC=4.08384, TT=1.365, SC=0.34207, ST=2.5092.

CCGAGGAGTCGATTACAGCATGAGTCAAACTGAGCTGCCTGAGTCCACTTCGCCAAGCGAGAGCTCGCCGGCCATGGCGTCCACCACGCCGGCGCCGTA
CGTCGTCGAGGACTGCGGCCCTAACCTCCAGCTCTTCAGCGACGGCACGGTGATCCGCTTCGAGGACTACAACATCCTCCCTCCGCCGGTCTTGCCTCCC
GCGCTGTCCACCGTCCAGTGGAAGGACGTCGTCTACGACGCCGGCCGCGGCCTCAAGCTGCGGGTGTACAGGCCACCGGCGGCTACCGTCGCCGGCGA
GAAGCTCCCGGTGCTCGTCTACTTCCACGGCGGCGGGTACTTCATCGGCAGCTTCGAGATGGATAACTTCCACGCGTGCTGCCTCCGCCTCGCCCACGAG
CTTCCCGCCGTCGTCCTCTCCGCCGACTACCGCCTCGCCCCCGAGCACCGCCTCCCCGCCGCCCACGACGACGCGGCGACCGCCATGTCCTGGGTGCGC
GACCAGGCCGTGGCGAGCGGGGACGCCGCCGACCCATGGCTCGCGGAGTCGGCGGATTTCGGCCGGGTGTTCGTCTCCGGCGACTCGGCCGGCGCGGG
CATCGTCCACCACGTCGCCCTCCGCCTCGGGTCGGGCCAGATCGCCGTCGACCCGGCGCGCGTCGCGGGGTGCGCGCTGCTGTTCCCGTACTTCGGCGG
CGAGGAGAGGACGAGGTCGGAGGCGGAGTACCCGCCGGGGCCGTTCCTGACGCTGCCCTTCTCCGACCAGGGGTGGCGCCTCGCGCTGCCGCGGGGCG
CCACGAGGGACCACCCGCTGGCCAACCCGTTCGGGCCGGAGAGCCCGGCGATGGACGCCGTCGCGCTGCCGCCGCTGCTCGTGGTGGTCGCCCAGCTC
GACCTGCTGCGCGACCGCGACGTGGACTACGCCGCGAGGCTGAGGGCGATGGGGAAGCAGGTGGAGATGGTGGAGTTCGAGGGGCAGCACCACGGGT
TCTTCGCCGTCGAGCCGTTAGGCGACGCCGGGAGCGAGCTGGTCCGGGTCGTGAGGCGCTTCGTCTACGGCAACGGCGGTGACGCCGCCGCCGCCGCC
GCCGCCGCGTCCAAGTAAATCCAGCCGACCAGACACCGATCGCTTTCTGTGGCATTGCGTCAGCTGCCACGTGACAGCATGAGACACGCCCCAGTTCCC
ACCATGTGATACACGCCCACGGCCCACCTTGGAAGCCTATTGGACATTGGATTTTAACTAGGCCCACTCATATACGACTTGTACCAAACCAATCTTCTCAA
AAAGAAAAGGAATAAGTTCACTCTTGGTCCCTCTATTTGTCACCCAGTCTGATTTTATCCCATGATCGCAAAACTGGGTACGACAGATCCTCCAACTTACG
AAACC>

TCONS_00090884; FPKM value: TC=4.56175, TT=9.12112, SC=2.40138, ST=32.6179.

TTCTCTTCTCATCATCTATATCACGATCGATCACAAAGAGGTTAACAGTAACACATGGGGTTCGGCTCGAGGGAGATGGCCTGCGCGCTCGTGGCGCTGGT
GCTCGGGCTCTGCTGCGTCGGCGGCGCACGGGCGACGGGGAGGATCGACGAGGGGCTGGAGGTGATGTGGGGCGACGGCCGGGGGAGCGTCTCGCCG
GACGGGCAGGTGATGGCGCTGTCGCTCGACCACACCTCCGGCTCCGGGTGGAGGTCCAAGAACACGTACCTGTTCGCGCGCGTCGACCTGCAGATCAA
GCTCGTCGCCAACAACTCCGCCGGCACGGTCACCACCTGCTACTTCATGTCGGAAGGGGAGTGGGATATCCACGATGAGGTGGACCTGGAGTTCCTCGG
CAACGTCACCGGGCAGCCGTACACGCTGCACACCAACGTCTTCGCCAATGGCACCGGCGGCAAAGAGCAGCAGTTCCACCTCTGGTTCGACCCCACCA
CCGACTTCCACACCTACTCCATCGTCTGGACCTCGCAGCACATCCTTGTGCTGGTGGACGGGACGCCGATCCGGGAGATGAAGAACCACGCGGACAAGG
GGATCGCCTACCCGTCGTCGCAGCGGATGCGGCTGTACGGGAGCCTGTGGAACGCCGACGACTGGGCCACGCAGGGCGGGCGCGTCAAGACGGACTGG
TCGCAGGCGCCGTTCGTCGCGCGGTACCGCAACTTCACCGCCACGGAAGCCGCGTCGTCGTCGTCGCCGGCAGGGTACGACCAGCAGATGGACGCCAC
GGCGCAGCAGGCGATGAAGTGGGCGCGCGACAACTACATGGTGTACGACTACTGCGCCGACAGCAAGCGGTTCCCGCAGGGCTTCCCGCCCGAGTGCT
CCATGCCGTAGAGTGGAGTGGAGACGCGCCAAAGCCTCGGTGCACTTTGATGAACGCGGACTCGATCGAACTCACGCAGGGCACGCATTGACCTCGTTT
TTTTATTTTTACTTCTCTTGATTGAATTGATTCATTCTTTCTATGTAAAGTAGATATATATATATATATATATATATATATATATATATGTATATGTATGCACGAAA
GATTTAAA>

TCONS_00091734; FPKM value: TC=2.15311, TT=5.16995, SC=0.143388, ST=3.82754

TAGCTGACCTGGGAGTCCAGTATATAAGTGCGTGTAGCCGCATGTACACAGGGTGGCCAAAACGAGACGTGCAGAGAGAGACACCAATCTCGTACGTAG
CATATCTACATCGCAGGAACACCCAAATCACTCGTGCGTAGGTATGGTTCTGACGCACGTCGAGGCGGTGGAGGAGGGCAGCGAGGCGGCGGCCGCCGT
GTTCGCGTCGAGGTACGTGCAGGACCCGGTGCCGAGGTACGAGCTCGGCGAGAGGTCGATATCCAAGGACGCCGCGTACCAGATCGTCCACGACGAGCT
CCTCCTGGACAGCAGCCCGCGCCTGAACCTGGCGTCCTTCGTCACCACCTGGATGGAGCCCGAGTGCGACAGGCTCATCCTCGAGGCCATCAACAAGAA
CTACGCCGACATGGACGAGTACCCCGTCACCACCGAGCTCCAGAACCGGTGCGTGAACATCATAGCGAGGCTGTTCAATGCGCCGGTGGGCGACGGCGA
GAAGGCGGTCGGGGTGGGCACGGTGGGGTCGTCGGAGGCCATAATGCTGGCCGGGCTGGCGTTCAAGCGGCGGTGGCAGAACCGGCGGAAGGCGGCG
GGGAAGCCCCACGACAAGCCCAACATCGTGACGGGGGCCAACGTGCAGGTGTGCTGGGAGAAGTTCGCGCGCTACTTCGAGGTGGAGCTCAAGGAGG
TGAAGCTGACCGAAGGCTGCTACGTGATGGACCCCGTCAAGGCCGTGGACATGGTCGACGAGAACACCATCTGCGTCGCCGCCATCCTCGGCTCCACCC
TCACCGGCGAGTTCGAGGACGTCAGGCGCCTCAACGACCTCCTCGCCGCCAAGAACAAGCGGACGGGTTGGGACACGCCGATCCACGTCGACGCGGCG

AGCGGCGGGTTCATCGCGCCGTTCATCTACCCGGAGCTGGAGTGGGACTTCCGGCTGCCGCTGGTGAAGAGCATCAACGTCAGCGGCCACAAGTACGGG
CTCGTCTACGCCGGCGTCGGGTGGGTCATCTGGCGCAACAAGGAGGACCTCCCCGAGGAGCTCATCTTCCACATCAACTACCTCGGCGCCGACCAGCCA
ACCTTCACGCTCAACTTCTCCAAAGGGTCCAGTCAGATTATTGCGCAATATTACCAGTTTCTTCGACTCGGATTTGAGGGGTACAAGAGCGTGATGAAGA
ACTGCATGGAGAGCGCGAGGACGCTCCGGGAGGGCCTGGAGAAGACGGGGCGGTTCACCATCATCTCCAAGGAGGAGGGCGTGCCGCTGGTGGCCTTC
ACGTTCAAGGACGGCGCCGGCGCGCAGGCCTTCAGGCTGTCGTCGGGCCTGCGCCGGTACGGGTGGATCGTGCCGGCGTACACGATGCCGGCGGCGCT
GGAGCACATGACGGTGCTCCGCGTCGTCGTCCGGGAAGACTTCGGCCGGCCGCTCGCCGAGCGGTTCCTGTCCCACGTCAGGATGGCCCTGGACGAGAT
GGACCTCGCCGCCAGGGCCCCCGTGCCCAGGGTGCAGCTCACCATCGAGCTCGGCCCCGCCCGGACCGCCGGCGAGGAGGCCTCGATCAGGGTGGTCA
AGAGCGAGGCCGTGCCCGTGCGCAAGAGCGTCCCGCTCGTCGCCGGCAAAACCAAGGGCGTTTGCTAGACCGGGTTAAATTTTTTTTAAAATACTGGT
GGGAACACGACCAAAATAAAATTTCAAAATGGGAGCGTACTAAACCTTCTTTATGCTACAGTAACTATGTAGTACAAGCATGCCGAAAGTTAATCATCGTG
TGTAATCGTATTAGAGTTTGCAACAGCATATTATAATGTGGAGAAGAACACGCAAGCGACATCAACCAGGACTCTGCACTTCTGATGAAGTTCTGAAACC
CATAAGGATTCAGATAAACATGAGCAGACACTGAAATATGTATGTACATAAAAAAATTTGTGCGAGGCATGCACATCATGCTTGAGCACGTTATTGTTGCAG
AAAACACGAATTCAACGAATTAAAGGGAAGATAACTTGCAT>

TCONS_00145395; FPKM value: TC=1.38808, TT=0.420934, SC=0.256135, ST=2.61443.

CTAACTCACTCACTCACTCCTTCTTCTTGCGACCTAGCTATCGAGCTAGAGCTTCCAAAACCCTAGCTAGCTAGCTGAGACAGTTAGTTTCATCTCGAACT
AGTTGTTGATATGGGCAGCTTGGACACCAACCCCACGGCCTTCTCCGCCTTCCCCGCCGGCGAGGGTGAAACCTTCCAGCCGCTCAACGCCGATGATGTC
CGGTCCTACCTCCACAAGGCGGTGGACTTCATCTCGGACTACTACAAGTCCGTGGAGTCCATGCCGGTGCTGCCCAATGTCAAGCCGGGGTACCTGCAGG
ACGAGCTCAGGGCCTCGCCGCCGACGTACTCGGCGCCGTTCGACGTCACCATGAAGGAGCTCCGGAGCTCCGTCGTCCCCGGGATGACGCACTGGGCG
AGCCCCAACTTCTTCGCGTTTTTCCCCTCCACGAATAGTGCGGCCGCCATTGCCGGCGACCTCATCGCGTCGGCGATGAACACGGTCGGGTTCACGTGGC
AGGCGTCGCCGGCGGCCACCGAGATGGAGGTGCTCGCGCTGGACTGGCTCGCGCAGATGCTCAACCTGCCGACGAGCTTCATGAACCGCACCGGCGAG
GGGCGTGGCACCGGCGGTGGGGTTATTCTGGGGACGACCAGCGAGGCGATGCTCGTCACGCTCGTTGCCGCGCGCGACGCCGCGCTGCGGCGGAGCGG
CAGCGACGGCGTGGCGGGACTCCACCGGCTCGCCGTGTACGCCGCCGACCAGACGCACTCCACGTTCTTCAAGGCGTGCCGCCTCGCCGGGTTTGATCC
GGCGAACATCCGGTCGATCCCCACCGGGGCCGAGACCGACTACGGCCTCGACCCGGCGAGGCTGCTGGAGGCGATGCAGGCCGACGCCGACGCCGGGC
TGGTGCCCACCTACGTGTGCGCCACGGTGGGCACCACGTCGTCCAACGCCGTCGACCCGGTGGGCGCCGTGGCCGACGTCGCGGCGAGGTTCGCCGCG
TGGGTGCACGTCGACGCGGCGTACGCCGGCAGCGCGTGCATCTGCCCGGAGTTCAGGCACCACCTCGACGGCGTGGAGCGCGTGGACTCCATCAGCATG
AGCCCCCACAAATGGCTGATGACCTGCCTCGACTGCACCTGCCTCTACGTGCGCGACACCCACCGCCTCACCGGCTCCCTCGAGACCAACCCGGAGTAC
CTCAAGAACCACGCCAGCGACTCCGGCGAGGTCACCGACCTCAAGGACATGCAGGTCGGCGTCGGCCGCCGCTTCCGGGGGCTCAAGCTCTGGATGGT
CATGCGCACCTACGGCGTCGCCAAGCTGCAGGAGCACATCCGGAGCGACGTCGCCATGGCCAAGGTGTTCGAGGACCTCGTCCGCGGCGACGACAGGT
TCGAGGTCGTCGTGCCGAGGAACTTCGCTCTCGTCTGCTTCAGGATCAGGGCCGGCGCCGGCGCCGCCGCCGCGACGGAGGAGGACGCCGACGAGGCG
AACCGCGAGCTGATGGAGCGGCTGAACAAGACCGGCAAGGCGTACGTGGCGCACACGGTGGTCGGCGGCAGGTTCGTGCTGCGCTTCGCGGTGGGCTC
GTCGCTGCAGGAAGAGCATCACGTGCGGAGCGCGTGGGAGCTCATCAAGAAGACGACCACCGAGATGATGAACTAAGTAAAGAGAAGATTACAAATAA
ATACACATATTTAGAACACATATACGTTTTTGATTTTTTTTTTTGGGGTTTTAGATGCGAATCTGTTGATTCATTTTGTGAGCTATTTATACCTATGGATATATG
GATCAAAATAAATTATTATTTGGTTGTAATTTTTTTAAAAGATGAACTGATACTATACCTTACACAAGAAATA>

TCONS_00054381; FPKM value: TC=0.846844, TT=1.72801, SC=0.286347, ST=2.75126.

GTAAGCTGCGCACCAACGCAGTTACGCAGAGCAGCCTAGCAGGTGCTGCGACCGCGAGCTTGAGAGCATTTTCATTTAAGAATCTCCCCCTCATCGATAA
ATAAATTCAAACAAAAAAAAGGAAGAAGGAAATTTCTGAAAAGGTTCGGCAGCTGAGCTCAGCTGAGCTCTGGTCCGAAGTCTTCTTCCCCCATCTTGG
ACGAAGCCGCGTTTTTCCACGGCCACCTCCCCCTAACATTCTCCCTCTTCTCTTCTCGCCGCGTTCAAAACCCACCTCCTCCTCCTCCTCCTCCTCCTCCA
ACCTCTCTCCCCACCTCGCGCCTTTTTCGCTATTTTCCTCTCTCTCCCAGCGCGCGGCCACCGCCCTCTTCTTCTTCCTGTTCTTCCACCACCACATCGGCG
CCGCCTGTCTCATGTGGGCTTAGCCATGACGCGGCTCGCCGTGCGCTCTCTGGTGGTCTTGTTCTTGACTGTGGCCTCGCCGTTTCTTGTTGCTGGTGGTG
GTGGCGCCAAGCTCAACGCGTCGTCGTCGTCGTCGCCGCTGTACGGGATCGAGTTCCCGCCGTTCAACGCCGGCGTCGCGGATGGCGGATGCGACGGGA
AGCTGATGGTGCAGGGGATGGAGGAGGTGAGCCGGTCCCCTTCGCTGAAGCTGCATATGACCCACCGGTCCGCCGCCGAGGCCGCCGCGGCGGGCAGG
ACGAGGAAGGAGTCGTTCTTGGATTCCGCGGGGAAGGACGTTGCCCGGATCCACACGATGCTCAGGAGGGTGGCCGGAGCAGGCGGAGGTAGGGCAGC
GACGAATTCGACGCCGCGGCGCGCGCTGGCAGAGCGGATTGTGGCGACGGTGGAGTCCGGCGTCGCGGTCGGGTCCGGGGAGTACTTGGTGGATCTCT
ACGTCGGTACGCCGCCGCGGCGGTTCCAGATGATCATGGACACCGGCAGCGACCTCAACTGGCTGCAGTGCGCGCCCTGCCTCGATTGCTTCGAGCAGC
GCGGCCCGGTGTTCGACCCGGCGGCCTCCCTTTCCTACCGCAACGTCACCTGCGGCGACCCCCGCTGCGGCCTCGTCGCGCCGCCGACGGCGCCCAGGG
CGTGCCGCCGCCCGCACTCCGACCCGTGCCCGTACTACTACTGGTACGGCGACCAGTCGAACACCACCGGCGACCTCGCCCTCGAGGCCTTCACCGTCA
ACCTCACCGCCCCCGGCGCGTCCCGGCGCGTCGACGACGTGGTGTTCGGCTGCGGCCACTCGAACCGCGGCCTCTTCCACGGGGCCGCGGGGCTGCTC
GGCCTCGGCCGCGGCGCCCTCTCCTTCGCGTCCCAGCTCCGCGCCGTGTACGGCCACGCCTTCTCCTACTGCCTCGTCGACCACGGCAGCAGCGTCGGG
AGCAAGATCGTGTTCGGCGACGACGACGCGCTGCTCGGCCACCCGCGGCTCAACTACACGGCGTTCGCGCCGTCCGCGGCGGCGGCGGCGGACACATT
CTACTACGTCCAGCTCAAGGGCGTCCTCGTCGGCGGCGAGAAGCTCAACATCTCGCCGTCCACGTGGGACGTCGGCAAGGACGGGTCCGGCGGCACGA
TCATCGACTCCGGCACGACGCTGAGCTACTTCGCGGAGCCGGCGTACGAGGTGATCCGGCGGGCGTTCGTGGAGCGAATGGACAAGGCGTACCCTCTCG
TCGCCGACTTCCCGGTGCTGAGCCCGTGCTACAACGTGTCCGGCGTGGAGAGGGTGGAGGTGCCGGAGTTCTCCCTCCTGTTCGCCGACGGCGCCGTCT
GGGACTTCCCGGCGGAGAACTACTTCGTCCGCCTCGACCCCGACGGCATCATGTGCCTCGCCGTCCTCGGCACGCCGCGCTCCGCCATGTCCATCATCGG
CAACTTCCAGCAGCAGAACTTCCACGTCCTCTACGACCTGCAGAACAACCGCCTCGGCTTTGCGCCGCGGCGGTGCGCCGAGGTGTAAGCCGGCGCCG
GCGCCGGCGCCGGCCACGAGAGGATCCACCAATTTGAAGGTCCTCATTTTGATCGAATTCGGGTTTAGGTGTGGCCAAATTTGGGGCAAATTTGGGTGTA
GAAATTTTGGGGGCGGAAATTAAAGGTCAGGAGGAGGGGGTTGACACGCAAAGTTGTATGTAGCACAAAGGTTTCGAAATCTCCAAGTAACAGAGATTT

TTTTAGGAATAATTAGGAATACTTTTTTACCAAAAAGATTGCAGTTTGTA>

TCONS_00002095; FPKM value: TC=0.0128545, TT=0.184657, SC=1.23957, ST=0.177896.

TTGAACGCTTCTGAACCTCTGATGTTTGATATGGTATCAGAGCCTTTCCACTAAACAAATCCCGATCCAATCTATGGCCAGCTCGTCGTCCAAGATGGCAG
CCACTAATCCTTTCGGAGGAAACACAATCTCAGAGAAATTAGCCAAAAACAACTATGCGCTATGGAAAGCGCAAGTCCTGGCGTCGGTGAGAGGAGCTC
GTCTTGAAGGGCATCTCACTGGCACCACAGCAGCGCCGGCCATCACCATCTCCGTGCCAGGCGAGAAGGAAGGCGACAAGGCCACCAGGGCGGCTAAT
CCCGCCTACGATGAATGGGTGGCTACGGATCAGCAGATCCTAGGGCTTCTACTGAGCACCCTGTCCAAAGATGTTCTGGCGCAGGTGGCCACCTGCGGCA
CTGCAGCTGCCGCCTGGAGCATGCTGGAGGAGATGTATACCTCCATGACAAGGGCACGCTTCATCAACACTCGGATCGCTCTCTCCAACACCAAAAAGG
GCGATCTCTCCATCACAGAATATGTCGCCAAGATGAGAGCACTTGGTGATGACATGACGGCGGCTGGCAAGGTCGTCGACGACGAAGATCTCATCTCCTA
CATCATTGCCGGCCTCGACGACACGTATGAACCGGTGATCTCCTCAATCGTCGGCAAATCTGAGCCGATGTCCTTCGGGGAGGCTTTCTCTCAACTGCTCA
GCTTTGAGCAGCGCAACAATCTCCGTCACGGCGGCGAGTCGTCAGCAAACCTGGCGAATCGAGGGCGCGGCACCACCGGCGGCAACGGAGGCCAACG
CGGACGCGGTGGGAACAACCGTGGACGCGGCGGCAATGGAGGCAACAACAGCGCAAACCGCGGCAAGGGTGGTCGCGGGAATGGCGGCTTCAATGGA
GGACGTCAAGGAGGTGGCGTCGACACTAGGCCCAAGTGCCAGCTATGTTACAAGCGAGGACATACGGTAATCAACTGTTGGTATCGTTATGATGAGGATT
TTGTTCCAGATGAGAAGTATGCAGGTTCTGCAGCCACCTCCTATGGTATCGATACCAACTGGTATGTGGATACTGGGGCAACTGATCACGTGACAGGAGA
GTTGGAGAAGCTCATCGTTCGTGATCGCTACAAAGGACATGATCAAGTGCATACCGCTAGCGGTGCAGGATCTAGCCACGAAGAAACTTCTGCTTAGGGG
GCCCTGCCGTGGTCGTCTCTATGCTCTTCCATCTACACCGCCACATGCACACTCAACCAAAGAAGTTTATGGAGTCTCCAAGCCATCTTTTGAAAGGTGGC
ACAGTCGTCTCGGTCATCCCTCCTCTCCAATTGTAGAGAAGGTCATTAGCAAAAATAATCTCCCATGTTTAGTTGAGTCTAATAAACAGTCTGTGTGTGATG
CTTGTCAAAAGGCAAAGAGTCATCAATTGCCTTATTCTATATCATCTAGTATATCTAGTCATCCTTTGGAACTTATTTATTCTGATGTTTGGGGTCCTGCCCCC
ACTTCTGTTGGAGGCAAACAGTATTATGTGAGTTTTATTGATGATTTCAGCAAGTTTACTTGGATTTATTTAATTAAGTTTAAATCTGAAGTTATCCAAAAGT
TCCATGAGTTTCAGGCTCTTGTTGAACGATTGTTTAACAGAAAAATCCTAGCGATGCAATCTGACTGGGGAGGAGAATATGAGAAACTTAATTCCTTCTTT
ACCAAAATAGGTATTACTCATCATGTGTCTTGTCCTCACACACACCAGCAAAATGGCTCAGCTGAAAGAAAAC>

TCONS_00058465; FPKM value: TC=0.0797721, TT=0.31375, SC=0.0183544, ST=1.77259.

GATCAACCTCGCTCGCTTACTCGTGTTTAGGCATGGACGTTTCTGCTGCGCTCAGCAGCGACTACTCGTCGGGGACGCCGTCGCCGGTGGCGGCCGACGC
CGACGACGGCTCCTCCGCCTACATGACGGTGTCGTCGGCGCCGCCCAAGCGGCGAGCGGGGCGGACCAAGTTCAAGGAGACGCGGCACCCCGTGTTCA
AGGGCGTGCGCCGGAGGAACCCCGGGAGGTGGGTGTGCGAGGTGCGCGAGCCGCACGGCAAGCAGCGGATATGGCTCGGGACGTTCGAGACAGCAGA
GATGGCGGCGCGCGCGCACGACGTCGCCGCGCTCGCGCTCCGCGGCCGCGCCGCCTGCCTCAACTTCGCCGACTCGCCGAGGCGCCTCCGCGTCCCGCC
CATCGGCGCAAGCCACGACGACATACGGAGGGCGGCGGCTGAGGCGGCCGAGGCATTCCGGCCGCCACCAGATGAGAGCAATGCGGCCACCGAGGTGG
CAGCCGCCGCATCGGGCGCCACTAATTCGAACGCCGAACAGTTCGCCTCCCACCCGTACTACGAGGTCATGGACGATGGGCTGGACTTGGGGATGCAGG
GCTATCTCGACATGGCGCAAGGGATGCTCATTGACCCGCCTCCAATGGCCGGTGATCCTGCCGTAGGTAGCGGCGAAGACGACAACGATGGCGAGGTCC
AGCTATGGAGCTACTGATCCTGCGCGTTTGAACTCAACTTGGTTTGGCGCGAAGAGATCGCATGTACAGCTTAAGGGAGTCGAGTACAAGTACCTCAGGT
GTACTCCACTCGTTGCCCCTTTCCCTTCCCTTTCGTTTTTCTTGAGCTTATCTGCAGGGTAATGTTATGTATTGCTGCTCTTCTGATGAAATGTGATCGGAAG
AAGCGGAAGGCCAGATCGAGCTTATGGGTTCTGAAGACGGTGAAGGCTTGTCGAGTGTTGTGAGCATATATTCAGAAAGTCAGGCACTGTGAAAGTATG
AATCAGATCAGCCTTGTTACGAATGAGAGTGATCGACCTTGTTCAGTGTTTATAATTGAACCACTTGTGTGTAATAAGCAGCAAAGCCATGTTGCTTGCTT
GATCTGACTCTTGGGAATGGTATATTTCTCAAAGAATGCAGGATTGACTACTCAGAATTTGACATTTTGCAGTGAAATGATAGGATTGTTAAATTAACATTG
GAGGAGAGGCATGTGTATATATGTTAAGAAACATTAGTAATGATGAG>

TCONS_00130099; FPKM value: TC=3.66869, TT=6.28368, SC=1.25617, ST=8.94166.

GCATATTTTGCGGTCCACCCCTTGGGATTTTGAGCTTTTACAGAATAGTCCGGACCCCTCCCCCGCCTATATATTTCCCGCCGCTCCGCCTCTCCAAACTCA
CCATCCATTCGCTTCTGTGTCTCTAGATACACTCGTCTCTGATCTCTTCTCGTCAGAGAGGGTTTCTTCTTTGAGCTCGTGATCTCTTCGAGATTTGTAGTAG
TACTACGTCGACGGTTAGGTGGTCGTCGCAGCAATGGCCGGCGGCGTGGTGGTGAACAACGGAGGGGGGAAGGACTACCCCGGGAAGCTCACCATGTT
CGTCCTCTTCGCCTGCATCGTCGCAGCCACCGGCGGCCTCATCTTCGGATATGACATCGGCATCTCCGGGGGTGTGACGTCGATGAACCCGTTCCTGATCA
AGTTCTTCCCGTCGGTGTACCGGAAGGAGCAGGCGGCGGAGAAGAACCAGAGCAACCAGTACTGCAAGTTCGACAGCCCGCTGCTGACCATGTTCACC
TCGTCGCTCTACCTCGCCGCGCTCGTCGCCTCCTTCTTCGCGTCCACCGTCACCCGCGTCGCGGGGCGCAAGTGGTCCATGTTCGGCGGCGGCGTCACCT
TCCTCGTCGGCGCCGCGCTCAACGGCGCCGCCAAGAACGTGCTCATGCTCATCCTCGGCCGCGTCCTCCTCGGTGTCGGCGTCGGCTTCGCCAACCAGTC
TGTGCCGCTGTACCTGTCGGAGATGGCGCCGGCGAGGCTGCGCGGGATGCTCAACATCGGGTTCCAGCTGATGATCACCATTGGCATCCTGTGCGCCAAC
CTGATCAACTACGGGACTGCCAAGATCAAGGGCGGGTGGGGGTGGCGCGTGAGCCTGGCGCTGGCGGCGGTGCCGGCGGCGATCATCGCCGTGGGCGC
GCTCTTCCTCCCTGACACGCCCAACTCCCTCATCGACCGCGGCCACACCGACGCCGCCAAGCGCATGCTCCGGCGCGTGCGCGGCACCGACGACATCGA
GGAGGAGTACAACGACCTGGTGGCCGCCAGCGAGGAGTCCAAGCTCGTGGCGCACCCGTGGCGGAACATCCTCCAGCGCCGGTACAGGCCGCAGCTGA
CCATGGCCATCGCCATCCCGCTGTTCCAGCAGCTGACGGGGATCAACGTCATCATGTTCTACGCGCCGGTGCTGTTCAAGACGCTGGGATTCGCCGACGA
CGCGTCCCTCATGTCCGCCGTGATCACCGGCCTCGTGAACGTCTTCGCCACCTTCGTGTCCATCGTGACCGTCGACCGCCTCGGCCGCCGGAAGCTGTTC
CTCCAGGGCGGGACGCAGATGCTGGCGTGCCAGATCGTCGTCGGAAGCCTCATCGGCGCCAAGTTCGGGTTCTCCGGCGTGGCCGACATCCCCAAGGCG
TACGCGGCGTTCGTGGTGCTCTTCATCTGCGCCTACGTCGCCGGGTTCGCGTGGTCGTGGGGGCCCCTGGGCTGGCTCGTCCCCAGCGAGATCTTCCCGC
TGGAGATCAGGTCGGCGGGGCAGAGCATCAACGTGTCGGTGAACATGCTCTTCACCTTCATCATCGCGCAGGCGTTCCTCCCCATGCTCTGCCGCTTCAA
GTTCATCCTCTTCTTCTTCTTCGGCGCGTGGGTGGTGATCATGACGCTGTTCGTCGCCTTCTTCCTGCCGGAGACCAAGAACGTGCCCATCGAGGAGATGG
TGCTCGTCTGGAAGTCGCACTGGTACTGGGGCAGGTTCATCCGCGACGAGGACGTGCACGTCGGCGCCGACGTCGAGATGCCCGCCGCCGGCAACCGC

AACGGCAAGGTCGACCCGGCCAAGCTCGCCAACTGATGACCTCAGCGCGTCGGTCGCTGACATCGTTCGCAAATAAGCTACCATTAAAAAAAATCTTATA
TTTCTTTTTTCTCATTTTTCTGTATTATACCCCAATTGTCTTAGTACTAGGACGACGACGAAAAAGCAGTCACTAGTCTCTCTTTCGTTTTCTTTTCATTTTTA
AAGTTCCGGTTTAATTTATCTGTGCCGGATACTCTACTTGGGGATTGTTTTGGATCAAGTATGAATATAAATG>

TCONS_00144852; FPKM value: TC=1.2716, TT=0.525426, SC=0.114359, ST=1.74968.

CTGCTCTGGAGTTGGCAACCAAAATGGAAGCCAACACCGGCGGCGGCGGCGGCGTCCTCGTCGTGTCACGCCGCATGATCCGGCCGGAGTTCAAGGAG
CTGCCGCCGGAGCATGATACGACGGTTCACCTTACGCCGTGGGATCTCCGGCTGCTCACCGTGGAGAACATCCAGAAGGGCATCCTCCTTCCCAAGCCAC
CCACCGGCGGCGAGACTCTCGTCGAACACCTGGCGTCGTCGTTCGCTCGCGCACTCGGCCGCTTCTACCCCTTCGCCGGCCGCCTCGTCGTGGAAGAGG
TGGATGGCGGAGCGTCGCCTGCGAGCAGCGTATCTGTCTCGCTGCGCTGCAACGACGAAGGCGCTGAGTTCGTCCACGCCGCGGCGCCCGACGTCGCCG
TCGCCGACATCGCCGCCTCGCTGCACATTCCACGCGTGGTCTGGTCCTTCTTCCCGCTCAATGGAGTGGTTGCTGCACACGCCGCGACGGAATCCCTGCC
GGTTCTGTCCACGCAGGTCACCGAGCTCTCTGACGGCGTGTTCATCGCCATGTCTGTCAACCACGTCGTCGGCGACGGCACCAATTTCTGGGAGTTCATG
AACACATGGTCGGAGATCAGTCGAAGTAGCGGCGGCGAACTGGGCATCTCGCCATCGGCGTCGACGTCGACTTCGCCGCCTCTGGTGGTCAAGAGGTGG
TTCTTGGACAACTGCACGGTGCCGATCCCTCTGCCGTTCGCCAAGCTCGAGCAGATCATCCCGCGAAGCGAGCACCAAGCGCCGGTGCAGGAATGCTTC
TTCGCTTTCTCCGCGGCGAGCATAAGGAAGCTGAAGGCGAAGGCGAACGACGAGATCGCCGGCGCCGCCGTCGCCATCTCCTCCCTCCAGGTGCTGCTC
GCGCTCGTGTGGCGCGCGGTTTCGCGAGCCCGCGGCCTCGCGCCGCGCCAGGAGACGGCGTACGTGGTGGTCGTCGGGTGCCGGGGACGCGTGGGCGG
CATCTCGTCGGGGTACATGGGGAACGCCGTGGTGCCCGGCGCGGTGAGGCTGACGGCCGGCGAGATCATGGAGAGGGGGCTCGGGTGGACGGCGTGGC
AGCTGAACAAGTACGTGGCGTCGTTCGACGAGGCGGCCATGAGGGGCGCGCTGGCGTCGTGGCCGCGGCGGCCGGACTTCTTCTCCGTCCTGAGCCTGC
TCGGCGGCGCCGCCATCATCACGGGGAGCTCGCCGCGGTTCGACGTGTTCGGCAACGACTTCGGGTGGGGGAGGCCGGCGACCGTGCGGAGCGGCGGC
GCGAACAAGTTCGACGGGAAGGTGACGGTGTACGAGGGGCCGGACGGCGCCGGGAGCATGTCGCTGGAGGTTTGCCTGACTCCGGCGGCGCTGGCGA
AGCTCGTCGCCGACGAGGAGTTCATGGGCGCGGTGACCACGCCATGAGTTGCATCCATGCATGCTTCTATTCATTGCGATTTGCGAGCTCAATTGGTTAAA
AAAAAACATTCACATCGACATCATGTAATCCATTTATGTCAATTTGTCAGTAGAAAGGTGATGAATTCATCAACAGCTATAGCTGATAGATCAGAGATATCA
CTGGATTACCTTTTGTATCAAACAAGGATAAATCAATATGTTTGTGAAGTGAAATTTCGAATGGTTTTTAGACGAATTTGAAGGAAAATTGAAGGATGCCC
ACGGAGGCCGAGGTCGTGGTCAGAAATGCCAGAGTTCAACCAGAGGATTCAAAT>

TCONS_00053041; FPKM value: TC=39.3722, TT=73.1821, SC=10.5781, ST=78.9446.

AAAGCGCTCGTTTTTAAGCGCTCCAGAGCCCTCCACGTAGTAATCTTATCCTCTATACTAACCTCTCTAGCTTCTTCAGTTCTTCTCCAAACCCAACCCCCC
CAAGAGGCCAAAACCCTCTCCTATATAGCCACACTCATCCTTGCAATTAGCTCAACTCAGCTCAAGTTGGCAATTCATATCAAACAGATAACATCAGTAGT
TCGCAGCTAGATCGATCGTCACAGCAACACAATCACCACGACGACGATGGCGGCGGCGGCGGTGGAGAAGAAGAGCGGGAGCGAGATGACGGTGGTGC
GGGGGCTGGACGTGGCGAGGTACATGGGCCGGTGGTACGAGATCGCGTCGCTGCCCAACTTCTTCCAGCCCCGGGACGGCCGGGACACGCGGGCGACG
TACGCGCTGCGGCCCGACGGCGCGACGGTGGACGTGCTGAACGAGACGTGGACCAGCAGCGGGAAGCGCGACTACATCAAGGGCACCGCCTACAAGG
CCGACCCGGCCAGCGACGAGGCCAAGCTCAAGGTCAAGTTCTACCTGCCCCCCTTCCTCCCCGTCATCCCCGTCGTCGGCGACTACTGGGTGCTCTACGT
CGACGACGACTACCAGTACGCGCTCGTCGGCGAGCCGCGGCGCAAGGACCTGTGGATCCTGTGCCGGCAGACGAGCATGGACGACGAGGTGTACGGGC
GGCTGCTGGAGAAGGCCAAGGAGGAAGGGTACGACGTCGAGAAGCTGCGCAAGACGCCGCAGGACGACCCGCCGCCGGAGAGCGACGCCGCGCCCA
CCGACACCAAGGGGACGTGGTGGTTCAAGTCGCTCTTCGGCAAATGATCGAGAAATTCTCAGCGGCCGATGGATGGATCAGAGGACGACGATGTATAGC
TATAGTATGAATTTGCCTTCAGTAGCAGTGGAGAATAAAATGGACTTTTGGTGTGAGTGTGTTCTTGAGGTGATTGTGTGTTTGTAATGCTGTGCTTTACAG
GAATTAATGGAAATGAACTCTTACA>

TCONS_00001933; FPKM value: TC=1.08435, TT=0.0583588, SC=2.55364, ST=0.974037.

AATCTACAAACATTAAATCTTTCGGGTTGTATATCTCTTGGTCACCTTCCTAAGGATATTAAAAACATGATTGGCCTTCGTCATCTCTATACTGATGGATGCAT
GTCCTTAAAATCCATGCCTCCAAACCTTGGGCATCTAACATCCCTACAGACACTAACATATTTTGTTGTGGGCAATAATTCTGGTTGTAGTAGCATTGGAGA
ACTAAGGCACCTCAAACTCCAAGGTCAACTCCAGCTATGTCATCTACAAAATGTAACAGAGGCAGATGTATCCATGAGCAGCCATGGGGAAGGGAAAGA
CCTAACCCAGCTATCTTTTGGATGGAAGGATGATCATAATGAAGTGATTGATTTACATGAGAAGGTACTAGATGCTTTTACTCCAAACAGCAGGCTTAAAAT
TCTATCTGTGGATTCCTACAGAAGTTCCAATTTTCCAACTTGGGTGACAAACCCCACAATGATGCAAGATCTAATCAAGCTCCAACTAGTTAGTTGCACAA
TGTGCGAGAGCCTTCCGCAATTGTGGCAATTGCCATCTCTTGAAATTCTTCATTTGGAAGGGCTGCAAAGTTTGCAATACCTGTGCTCGGGTGTTGACAAT
TCGACATCTTCAACGTTTCCTAAATTAAGGGAACTTATTTTAGTTGATCTAAAAAGTTTGAATGGTTGGTGGGAAGTAAAAGGAGGACCTGGGCAAAAGC
TAGTGTTTCCCCTTCTAGAGATTCTATCAATTGATAGTTGCTCTAACTTGGAAAACTTTCCAGATGCAGTAATATTTGGTGAATCTTCTCAATTCTTAGATAAT
AAAGGGAACTCCCCATTTCCAGCTTTAAAAAACCTCAAGTTGCACAATCTGAAGAGTTTGAAGGCATGGGGAACACAAGAAAGATATCAACCAATATTT
CCTCAACTTGAGAATGCTAATATTATGGAGTGCCCGGAGTTGGCAACTCTACCTGAAGCACCAAAGCTGAGAGTATTAGTATTTCCCGAAGATAAATCATT
GATGTGGTTATCCATAGCAAGATATATGGCAACACTGTCCGATGTCAGATTAACAATTGCAGCTTCTTCCTCACAAGTACAATGTGCAATACAACAGGTCA
GTGGTACAGAAGAATTTAGTCATAAAACTTCTAATGCAACTATGGAACTACGGGGATGTTACTTCTTCTGTATGGATTGGGAATGTTTTGTGAACCTGCAA
GATTTAGTAATCAACTGCTGCAACGAACTCGTCTATTGGCCACTGAAACAGCTCCAATGCTTGGTATCCTTGAAAAGATTAACAGTTTATTCTTGCAATAAT
CTGACTAAATCTGGTGATGTTCTAGAAGCGCCATTAGAAAAAAATCAGCTACTCCCATGCTTGGAGTATATAGAAATAAAAGATTGTCCCAAATTGGTTGA
GGTTCTCATCCTTCCTTCATCTCTAAGGGAAATATATATTGAGAGATGTGGTAAGCTCGAGTTCATATGGGGTCAAAAGGACACTGAAAATAAGAGTTGGT
ATGCTGAAAACAAAGATGATTTGAGGTCAGAATCTTATAGCATCCTAGTATCCTCTGCAGATGCACCTTTGGCAACAAATACACATTTACCTTGTATGGAGT
CTCTAACAGTGATAAGTTGTCAAAGCTTAGTAGTGCTTCTCAATTTTCCCCTATATCTGAAGGAAATACACATTTGGAGTTGCCCTGAGCTTAGATCTATAA
GGGGAAAGCAGGATATAAAAGTCGAAAGTAAATATGTTGAGCGTAATAACGGCATGGCTATATCAGAATCTAGCAGTGATCTCAGTGCATCTATAACTATA

GAAGATCAAGGAACATGGAGAAGCAAATATCTCCTGCCATGCTTAGAATACCTAAGAATAGCATATTGTGTAAGCTTGGTAGAGGTTCTTGCTCTTCCTTC
ATCCATGAGAACTATAATTATTTCAGAGTGTCCTAAGCTTGAAGTCCTGTCAGGGAAGCTTGATAAACTTGGGCAACTAGATATTCGATTTTGTGAAAAGC
TGAAACTAGTGGAGTCATACGAAGGATCCTTCTCATCATTGGAAACTGTCTCCATTGTCGGCTGCGAGAACATGGCATCGTTACCAAATAAACATTCAAAT
ACTCCCTGTACAAAAGGTAGTGCATGATTGAATCCTTTCTGTATTCAAGTCAAGAGGAATTTCATAATGTCTATGGGGGCCGAAAACAACTTATTTTTGCAT
CAGTAATTTGTATCAGGGATTGTTCTCTTCAGAGGTACAAATATATGGAGATATCTACTCGGTACTGTCTCAATTTTCATTTTCCCTCCAAATGTGGACCATG
ATGTGGACTATGGAAAACCAAGAACATAATCTTTCTTCTGCTGAATTCTTCAAATTTGTCTAGGTAAATGACGTTGACATATATAAGCATGTGGCTATGGAA
TTGCTGCCCATAAGAGAAGTTCACAGGACAGCAGCGCAATAATGGTACGATTGATAATTGCACTCGTGCTGTATGAATTTTGCACAGTTTTGCTTAATGGG
AGTGTCCCATTCTTAAAGCTTACTAAAGTCTTGTTTGGCACAACTATCGAAGGTTTTTAGGAGCTAGGTATTTCTAAGTCCACAAATTCATTAATCTGTATCT
GCG>

TCONS_00115693; FPKM value: TC=55.5265, TT=25.0189, SC=8.83393, ST=20.705.

TCTCAACAGAGCAACACAAACTCACACATTGCCAATTCCCTTTCTTGGTTTGATTCTGGCTGCTGCAATGGCTTCGTCGCTGGCCCTGGTAGCTCTGCTCC
TCGTGAGCTGCGCCGTGGTGGCGGCGGCGGCGACCAAGTACACCGTCGGCGACACGTCCGGCTGGGCGATGGGGGCGGATTACACCACCTGGGCCAGC
GACAAGAAATTCAAAATGGGCGATACCCTCGTGTTCAACTACGCCGGTGGGGCGCACTCGGTGGATGAGGTGAGCGCAGCGGACTACGCTGCGTGCACC
GCTAGCAACGCGCTCCAGAGCGACAGCAGCGGCACGACCACCGTCACCCTCAAGACCGCCGGCAAGCACTACTTCATCTGCGGCATCGCCGGCCACTG
CAGCAACGGCATGAAGCTCGTCGTGGACGTTGCCGCGGCCTCTCCAGCCCCAGCCCCAAAAGCCCCGTCCACCACACCGACGACTCCATCTACGACACC
AGCGACACCGGCGTCCCCTGGCACAAGCTCCGGTCTGACGCCCACAACCCCAGCAACGGTGCTCGCGCCGCCCGCCAAGCAGTCCGCCGGTGCAGCTG
GGCTCAGGGCCAGGTCATGGGCTATGCTGGGCCTGGCAGGACTTGCCGCCGTGCAACTGGGCCTCTTCTAGGGCCAAGGCCAGGGTGGAGGCAGTGCT
GTTAGGTTGCATGTCATTTAGACATCGACACACACCGTGTCAAGTGTTTGTTCAGGAAGTGAGTTGAGGTTTCTTTTCACAGAGGGAACTGGTGCTTTTG
TTGTGGTGAGCTGGCAGTAGTTGGATGCTGTGTTTGAATTGACTTCCTGTTAATTTGTTGTCCTATCTATGTCATGGATTTTTAGC>

TCONS_00032876; FPKM value: TC=2.136, TT=4.14376, SC=0.63361, ST=4.77596.

GAAGATGTGGCCGTGCGACTGCTTGTTACGTGATGAATAGCGAAATTCTACCCAAAAAAGAGAGAAAAAACAGCTGTGTGCTAGAGAGATATAAGGAGA
GAGGCACCATTGTGCTCTTACACCAGCACAACATGTGCTGTGAATTTGAAAGGGGAAATACCAGCTATGGAAGCAGCGATAGTGAGTGCTTCGACGGGA
GTGATGCGTTCCCTCCTCGCCAAGCTCACAAACCTTCTTGGTGGTGAGTACAAGCTACTTAAGTGGCTCCGTAGGGAGATGGAGTTCCTCGAGAGCGAGC
TCCGTAGCATGAGCATCTTCTTGGAGAGGTTGGAGGACACGCAAAAGCTTCACCCCCAAATGAAGGATTGGAGGGATCGAGTACGCGAGTTGGCATACG
ACATTGAGGATTGCATCGACGACTTCATACTACAGCTTGATTCCAAGGATGCGAAGGTTGGGTTTGGGCAAAAGCTTCTTGCATCCCGTAGGATTGGCCAC
ATGATTCGAGAACTCAAGGCGCGTGTAATGGAGGAGAGTGAGAGACAAAGGAGGTACATGCTTGATGGCTTGGCATCTGGGCCTAGTGTTCGTGTGAAG
GTTGATCCACGGCTATCGGCTCTCTATGTCGACGAGGACAGATTAGTTGGTATTGATGCCCCGAGAGATGAGATCATTGGGCGGCTGTTGGACAAGCGGA
GAAGTGCCTCAGCAAAACAGGTCATGACAATTTCCATCGTGGGTTGCGGAGGATTGGGGAAGACCACCCTTGCCAATCAAATTTACTGCAAAATCAAAG
GAAAATTTGAGTGCGCAGCTTTTGCGTCAGTGTTCCAAAATCCCAACACCAAGAAGGTTTTGACGAATATTCTTTCCCAAGTGGCCACTACTGCTGCTGT
GGAAGACGATGAGCAAGCCATCATCAACAAGCTAAGGGAATACCTTAGTGACAAGAGGTACATTGTCATAGTTGATGATATATGGGATATGCAAATATGGA
AATTTATTGAATGTGCGTTGGTCAAGAACTGCCGTGGTAGCAGAATTATCACAACAACACGTATACATGATATTGCTAAATTGTGCTGTTCATCTCATGGCG
ATTATATTTACGAAATGAAGCCTCTTGGAGTTATTGACTCCAAAATATTGTTCGACAAAAGAATTTTTGATCCAGAGGAAAGGCGCCCACCGCAGTTGACA
GAAGTTTCTGAGGAAATATTGAAAAAATGTGGTGGTTTGCCTTTGGCGATCATTTCCATATCAAGTTTGTTGGCAAGCAAACCAAAGTCAAAAGATCAAT
GGGACAGGGTCAAGGTTTCTCTTAGCTCTACGCTTGAAAGGACTCCAGACATTGAAACCATGGAATGGGTGCTATCACTCAGTTACTCTGATCTTCCTAAT
CATTTAAAAACATGTCTGTTATATTTGAGTATATTCCCTGAGGGCTATGAGATAAACAGGGAACGTTTGGTGAGTAGATGGATTGCTGAAGGATTCATTTAC
AAAAAACATGGACAAAATCCATATGAAGTGGGGGACAGTTATTTTAATGAGCTTGTGAACAGAAGCTTGATTCAACCAGCAAACATTAAGCCTGATGGTC
AGACAAATGCTTGTCGAGTGGATGACACCGTTCATGATTTTATTGTGTCCATGTCAGTTGAAGAAAATTTCGTTACATTATTTGGTGGTTCAAAATTGGTAC
CAAGGTCACATGGTAAGGTTCGTCGATTATCTATTCAAAATGGCGGCATTCAAGAAAATATTGTCACATCAACGCACTTGGTTACATCGCAAGTCCGGTCA
CTAACTTTATTTGCTGTAGAAATGCCATCTCTTTTAGGTTTTGGTATGTTACGTGTATTGGACCTAGAAGATTGTTATGCATTAGAAGACCATCATCTTACGA
ACCTGGAAAGGCTAGTCCAGTTACGGTATTTGAGTATAAGAACATCACCCATTAGTGAACTCCCGAAACAAATTGGGCAGCTTCAATATCTGGAAACACT
AGATTTAAGAGCTACGGGTGTGGAAGAATTGCCATCAACCATTGGTAGACTAAAGAGTTTGGTCCGTTTATTCGTTGATTATCATGTTAAATTACCAAAAG
AAATCAGCAATATGCATGCACTGGAAGAGCTAACAAGTTTCTCAGCCTTGATGTACTCTCCAGACTTTCTGAAAGAACTAGGTCAGCTTACAAACATGAG
GGTGCTCCGAGTAATTTGTGACTGTGATAGCTTTAAAGGTGATGCAGGAAGTTGCTTGGAAAATTTAGCTTCTTCTTTATGTAATCT>

TCONS_00003660; FPKM value: TC=12.8229, TT=1.39232, SC=11.3015, ST=1.07173.

GGTAAGCAATCTAATCCAGTCATGATCCTCGTAATTACACATTGCCATTCTTAGCAAAGCAAAGATACGCTCCTAATAAGCATCCAATCAAACCCAGCAGAT
TATGGATGGGTTTATCCCTTGTAAAGATTAGCTTCTAAACTAAGCCAGCCATTTCGCCATCTATAAATACTTAGGAGGACCCCATTGTATTCCATCAACACAG
AAGACAAACAAGGTCCAATTCATCACAAAAATACACATACACACCACACATACTTTTGCAGAGCAGCAACATGAAGTTCAGCGCCATTTTCATGCTGTTC
CTCGCCCTCGGAATCCTCGCCATCGCGGTGCACGGTGGCAGGACACACGCCATCATCGAGCCGGCGAAGAAATCGGCTGCTGCTGGCGTCGCGCAGCCG
CAGTTCGACCCGATCACCATCTGCAGCCCGTCCAACTTCTGCATCCCGGAGGCGTGGAGCTCCTGCTACAGGTGCATCGTGAAGCCCGACGACAACCCG
CCCTTCCGAACCATCGACGAGTGCAATAGCAACTGCCCGGTTCCTCCGGCCAACGCTTAATTAGTTACGTGTTCTGTTGGGTGAGGATGGTGTCTTCTTGA
TCTTTAGCGATAACGGGGGTGCACCCATGTGATCCTATACGGCTAT>

TCONS_00052789; FPKM value: TC=15.0243, TT=7.73985, SC=3.83416, ST=12.023.

CTCGATTTTTGCTAGCACGCTTTTCATAGTGCTAATCAGTATGTTTTGTGTGAAAATTTTATGTATAGAAGTTACTTTAAAATATAAAATAAATTTCTTTTTTA
AGTTTATAATAATTAAAACTAAATTAATCATAAGGTATTAGCTATTTTGCTTTGTATGAGTATAATAGTCAAATCAAAGTACTCATCCATAGTGCACAATCATT
TAAAGAGAACCAAATGAGACCCTCTCTCTCTATGCTCATTTCCCCCCTATAAATACTCTACTTGCCCAATCCCTCTTTCCCCACAAGAACCACATCCTCTCA
TCTCTGTCATTCTGCAGCTAGCTGAAAAAATCTTCAGAGAATCATCAGTTCATCACAAAAGACTATCAAGCGAGGCAAGTACGTGACGGTGACTGTGATG
AGCGGCGGCGGCGGCGGCGGGAGGGGGATGACGAGGGGGCTGGACCTGAAGCTGAACCTGTCGCTGCCGGCGGTGGCGCGCGCGGTGTCGCCGGCGG
CGGACGACGAGTCGTCGCCGAGCTCGTGCCTGTCGTCGGAGAGCGAGCTGCGGCAGCAGCACGGCGGCGGCGGCGGGCAGCTGCAGTGGTCGGACAG
CCCGGAGGCGACGTCGATGGTGCTCGCCGCGTGCCCGCGCTGCTTCCTCTACGTCATGCTCGCCGAGGCCGACCCGCGCTGCCCCAAGTGCCGGAGCCC
CGTCATCCTCGACTTCCTCCACGCCGGCGGCGGCGGCGGCATCAACGCCGACGGCCGCCGCCACCGGAGGGGATGAACATGGCCATGACCATGATGGCA
GCAAGAGATCGAGGAGAAGCATCTACACGCTTCTTTGATCGCGCAGAGGCATCAGGAATTCAGGATCAAGAAGAACAACGAGAAGAAGAAATCTCACC
TTGATGATGATCACAGTTAATTAGTTCTAGGCCCCATTAGTTTCTTCTCAATTAGCAGCAGTAGTATCATGATTAATCAGCTCGATCCTCTGCAGATTATGCA
GTATAACCATGATT>

TCONS_00053233; FPKM value: TC=0.812947, TT=5.65179, SC=10.6392, ST=3.70345.

GGATTAGCTTCCTAACTTTGAGGGATAGATCATGGTTGTTAGTCTCCTGTGTTTATCTCTTAATCTTGATAAGATTTGTTGTAATCTCTCCGCTGTAATCGTGC
CACGCCAGGGGCTGTTCCTGTGCATATTTAACACGCAACCGTGGAGGACCGAGAGAGGTATCCACGTTAAATCCAATCTTTTCATGGTATCAGAGCCTGCT
TCCTCGACGTCGTGATTATAGCGTCGTCTTCATCCTCGCTAGCCGGTGGCGCTGCTCCTTCTCTCGCGCACGCGGTCACAGAGAAACTCTCCCGCAGCAA
CTATCCTCTCTGAAAGGCGCAAGTTCTTGCTGCGCTCAGGGGAGCTCAGATGGCTGGATACATCGACGGCACAAAGGAGGAGCCGCCGGCCACCATCAT
CACCAAGAAGGACGACAAGGATGTGCGGGTCACCAATCCCGCGCATACCGAGTGGGTGGCGCAAGAGCAGCAGGTTCTCAGCTACCTGTTGCTCTCCCT
CTCACGTGATGTCCTCATCCAGGTGGTCTCAATTCCCACTGCTGTTGGGGTTTGGGATGCCATAAGTGGGATGTTTGCATCTCACTCCCGTGCGCGTGTGA
TGAACACACGCATGGCTCTGACGGCCACGAAGAAGGGCAATCTGAAGGTTGCCGAGTACGTGGCCAAGATGCGGGGTCTTGCTGATGACATGGCCTCGG
CAGGAAAGAAGCTCGACGACGACGACATCGTCTCCGACATCCTGGC>

TCONS_00075473; FPKM value: TC=5.82103, TT=10.99, SC=1.06504, ST=7.82294.

GGCCGATCCAATTTGGAGTTGGTATGGGAAATTTGTGTGGGCTATAGTTTGTGCCATGTGAAATTGGGCTTAGCCCAGAAGTAAGTATGAGAAAGGGAAG
GAGGAGGGGAAGAAAAATCTGAGAAGGAGCCAAATGGAGAGCTAGTAGGTCCAAAGGGTGTGGAGAGGAGAGGTGTAAGAAGAAATCTCTCTCTTTTT
TTGAGATGGGAGGGAGGAAATCTATCTCTGAATGTGAGCGAAGCAAAGGATTTCCCTAGCTCCATCTTCGTCCTCGTCCACACAAAATGTTTTCCTGTTTG
TTCGTTTGTTTACCTGATGCTGCTGCTGCTGCGGCCATTTGCACATCTCTCTCTCTCGCTTCTGCCACTGCTGGCTGTGCTGCTTCCCTAGCTCCTCGGTTG
GATCAAAGAGAAGAGAAGAGAAGAGTTAATGCTGATGGCCTCCGAGGCGTGGCTGTCGCCGGCTTCCCGCCTCTTCCACCCTCCCCGCTCCTCCACCGC
CGCCTTCTTCTCCGCTTCCGGGCTCCCCCGCCTGCCTCGCCGCTCCTCCTGTGCACTCGCAACCTCCACGCGTGGGCGGCGCCTCCGCCTTGGCGCCACC
GATCAGCAGCAGCAGCAAGGCAAACAAGATGGTGATGAGGTTGTGGACAGCAACGTGCTCCAGTACTGCAGCATCGACGGGAAGGGGAAGAAGGCCG
AGAAGAGGTCGCTGGGTGAGATGGAGCAGGAGTTCCTTCAGGCTCTTCAGGCCTTCTACTACGACCAGAAGGCCATCATGTCCAACGAGGAGTTCGACA
ACCTCAAGGAGGAGCTCATGTGGGAAGGCAGCAGCGTTGTCATGCTAAGCCCTGACGAACAACGCCTGCTCGAAGCCTCCATGGCCTATGCTGCCGGCA
ACCCCATCATGTCTGACGCTGAATTCGACCAACTCAAACTCAGATTGAAGAAAGACGGAAGTGACATTGTCACCGAAGGTCCCAGGTGCAGTCTACGCA
GCCGAAAGGTGTACAGTGATTTGACCGTTGACTACCTAAAGATGTTCCTGCTAAATGTTCCAGCAACTACTCTTGCTCTAGGACTGTTCTTCTTCATCGAT
GAGTTGACTGGCTTTGAGATCAACATATTCCAGCTGCCAGAGCCTTTCGGTTTCATTTTCACATGGTTTGCTGCTTTGCCTCTAATATTGTTCATAGCACAG
TCGATAACTAATGCCATAGTGAACGACTTCTTAATCCTCAAGGGGCCATGTCCGAATTGTGGCGCTGAAAACCTTTCCTTCTTCGGGACTATCCTGTCAGT
GTCTAGTGGCGGTGAAACAAACAAAGTGAAATGTGCAGATTGTAGCACGGAGATGGTATATGACTCGAAATCTCGGCTCATCACCCTTCCAGAACCATCG
CAATGAAATGAATGGTGAATTCTTCAAGGTACGATGCCAGATGGAGAGATGCAAACAAGGAGGTGATCAGGTAGGAAACAACAAATAAAATGGAATTAT
TGTAACGGATGGGTGTTCACAAATTGCATGCAGAG>

TCONS_00108299; FPKM value: TC=4.53854, TT=10.2554, SC=0.109454, ST=1.86374.

TCGTCGTGCATGCCACCCCACTAGCTACCTACGTCGTACACACCCCAACGTGCATGCCACCATCACCAAATCCCTTTCTCTATATTAGACGTACGCTAATCC
CATGGATGATCACAGAATTATATCCAAGAAAACCAACTGACCAAGAAGAAACTCTCCTCCTGATCACAGAATTATATGCAAGCCTCGTCGTCGACGCGCC
CCCTGGCTCCCTCCTGCGTGGCGTGCGGCCTCCTGGCGCCATGCCGGAGAGCGCTCGCGCGCCTCTTCCGCATCCCGGCGTCCGCCGCGCTCTCCGTCAG
GGCCTTCCGCTTCCGCACCCTCCGCAGGGCCGCCTCCAAGATGAGCCCGCGCCGCCACCGCCACTGCCGCCACCCACGACGACGCACGTTCCGGTCGGT
CCGCGCGGTGTTCTGGCCGCTCGTCCCGCCGCCAGCTACCACTACTGCGTCCTCCACTGATCAGGGCGAGAGCGCCGCCGGCGCCACCGCGCGGAGCGT
GGTAGCACCGGAGGAGGAGGCGGCGGCGGAGGAGGAAGAAGCGGCGACCGTGCTCGCGCCAGTGCCGTCGCCGGAGACGCCGGCGTACGTGAAGATG
GTGGCGCGGCTGCGGTCGAGGAGGAGACGCGGGGAGGAGGAGGAGGGGCCGTGCCGGAGCTTCGAGGAGCGGCTGATGGAGATGCTGCTGGAGGAG
GGGAAGGTCCGGGACCAGCAGGACGTGGAGGAGCTCCTCCGGTGCTGGGAGCGGCTCAAGTCGCCGGTGTTCGTCGAGCTCGTCTGCAGGTTCTACGG
CGAGCTGTGCAAGGATCTCTTCTCTCCCGGCGAAGAAGACGGCGGCGACGAGGGCGCCTCGTCCACAACCACAATCACACCGGCTGGCGACTCTAGTTA
GGGTGATATGAGAGTGATCATATTGATGGTGATGAACTGATGATACATCATATACCACCTTGTATTTATTATTACTAGAAAAAAGGTCGTGCGTTACAACA>

TCONS_00116758; FPKM value: TC=5.95166, TT=10.61, SC=2.42293, ST=21.1886.

GTTGACGGCAAGGAACTCGCGCGCGAAGCCGTCGTGAGATCCACGCGACCACCCTGGCTTTTGCACTTGCAGAGTTGCAGTCGCGTGCGTCACGCGGC
GAGGTGACCAGGTCGCGTACGCTCGCGTTCTTTCGGCCGCCTCGCCTTCCACCGCGTCCGCGAATTTTCTCGACCCATCTGTCCAAGAAAACACACAAAT

CCACTTCGATTACAACACAAATCCTCTCCCCCCCACGAGTATATATAATCCCCGATCTCTCTCCCCCCCATTGGCAACCTGTGCAAGCAAGGCAAGCAACC
ACAAATTCGAAGCAAAATTCAAGCCTACACGCCAGGTTTTTCGTCGAGAGGTTGTTCGATCTCGTCGTCTTCGTCGGCGGTGATGGACAAGGTGCTGGCA
TTCTCGATCCTGAGCGCGTCGCCGGCGGACATTGCCGCCGGTGGCTACTGGGCTCGGCTGTCGTGGCGGAGGAAGGCGGATGACCAGGCGGTGGATGGC
CGGAGGCAGCCGTCGGAGCAGCAGCAGCGCGGGGAGGGGAGCTCGCCGTCGCAGAGGGAAGAGCGGCGGCGGCCACGCGAAGCGCCGCCCCTGCCA
CCGCGGTTCGCCCCGGAGTTCGACGGGATCGACTGCTTCGAGACCATCGTGATGCATTAGCCATTGCGAAATCATCATTATTACTACACGAGAGGATTTGA
TCTGACACGGGGTACTAATTTGGTGTTTTTTTAAAAAAATTTCACACGAACATGTATACGATGATTACTAGTCCCCTAACTATCCAGCTTCTATCTGTTGTTT
TAATTGAGATGACGGGTTGTACAGAGCTGGAAACTAATCGAATATGAGCCCCTCTTCTTTTTTTCTGAAATCAAATTGAATTGTGTACTGAATTTGACCTGC
TTTGTTTGATTTAGC>

TCONS_00140366; FPKM value: TC=2.45959, TT=5.02738, SC=1.36316, ST=34.3699.

CGAGCCACGCGATGCCAACCGTGGACGCGGCGGCGCGCGACGCGACGCGGTGCCGCCTTTCTTCTCCTGCCGTTCTCACCCACCTCTCCACGAACAGAC
ACACACTCACCTGCCTATATAACCATGTGCCCAGCTCACCTGAGCTCGCTACCCACCCACCCATCTCATCTCATCATCGCCTTTCCTCCGATTCGATTCACC
GGTCGAACAGATCGAGCTCACTCAGCTGTGAGGTGAGTTTCTTGGATTGGATCATCGATCGTCGTCGTCGTCTATGGCGGTGGTGCAGATGCTGTCGTGG
CTCAGCTCCGGGGCGGCGTGGTTCGTGTTCCTGAACGTCATCGTCGGCGCCATCGCCGTCGTGTCGTGGCTGGGGCAGGGCGTGGGCGGGACGACCTCG
ACGCCGTCGTCGGCGGCGCGGCGGAGGCTGGCGCGCACCGCGTCGACCGTTGTGATGGAGAGGCTCCGGTCCGTCGCCAACTTCCCCTTCCAGTACCTC
TCCGGCGACTACAGCGCCACGCCGCTCCACGTCCACGGCGACGTCTCGTCGGGGTCCGATTACTTCTACTACCCGCGTGAGGCAGAGGAGGAGTTGGTG
GAGGCCGTCGCGGCGGCGCCGTTCCGGCCGGAGCCGCCGGCGCCGGTGAGGGAGGTCGTGGCGGCGACGACGCCGTCCATGGCGGCGCCACGGAGCT
CGCCTGTGGCCGCCGCAACAGCGGCGGCGATCAAGAACGAGGAAGAAGAGGCGGAAGCAGAGGAGGAGGAGGAGTCCATCAGCTTGGACGAGGCGT
ACGCGCTGGCCCAGCAGGCGCAGGCGCGCTCGCCGCGTTCCGCACCAGATGCCGCGGCGGCGGCGAAGGCGGCGGACGCCAAGCCGCGGCGGGCGCG
GGCAGAGGAGGTAGAGGAAGGGAAGGCGGAGGTGAACGCGCGGGCCGAGCGGTTCATCAAGCAGTTCAGGGAGGACCTCAAGCTGCAGCGCATCAAC
TCCATCATCAACTACACCAACGCGCTGCGCCGCCGCGGCGCCGGCGGCGTCGCGGCGACCGCGCCGGCGCCGGCACGGTAGCCGCCCGCGTTTCGCTGC
CGCTCCGATCCACTCTACACGATCCTTGTCGCGTGGTTTCGCTTGGTTAGATAGGGGATTTGTTTGTTCATTGTGACGGATCAACGCTACAACACACAACA
ATTTTGGATTGTTTACTTATACTTGTAGTACATACTACTACAAAATTTGTGATTCAAATATGATTCAAATGCTGGGACATGTCTCGTAGCTCAAGATCAGAAG
CGTTTGGTGACTCAGTTTGCGATCCATCCGAAGGATTTTCGATCTTGGATCTAGGCTCGTTGAGCTGCTTTTATTATTTGTGATTGCAAGATCGAATGAATA
GAAGATGGCGTCGATCGAGCCGAAAATTAATCTGTTCTTGTGCCAGCAGGGGATGTCGGGTTGTGCGGCTGTGCAATAGTAGCAGTAGTGGAAAG>

TCONS_00152703; FPKM value: TC=13.1683, TT=25.1749, SC=2.58101, ST=23.2902.

GTTGAGTTTAGAGAAAAGTTAAGGTTTCAGGTGTTTATGTTGTTGTGTTTTGTAAACATGAATAAAAGTAATAAAGAGAGATCCTGGATTTTTATTTTCCTA
GGTGCAGTTTTCTAACCGGCGGAACATCGGCAGTCAAACCGACGACTCCACGGCGGTCAGAACGGTTGCGGCGACAGGCGACGATAGCAATATCGGAC
GGTTAGATCGAAGCTACTACATCGGTCAGACCGGCGTATCTATTCCGGTCAAACCGATCTCCATCAAAACAAAGCGCCGCACCCCTGCGTGCGAGACGGC
GTCGCGTCGCTCGCCACGACGGACATGCAGCACCTGCACGGCGACGTGCTCGAGTCGGCGGTGGAGCGCGTGCCGGCGCCCGATCTCGCCGCCGCGGC
GCTGGTGTCACGGGAGTGGCTCCGCGCGGTGCGCGCCGCGCTGCGACGGCGCATGCTGCGGCTGCCGTGGCTCGTCGTCCACGTGATCCATCTCCGGGG
CCAGCGGCGCCTCGCCGCGGCTTACGACCCGCGCTCCGGGGCGTGGCTCGCCGTGCCCACGGCGCCACCGGCGCGCCACGGCGCGACGTCGCCGCCGC
AGCCGCACTCGCACGTCCGCCTGATGCGGGGCGCGAGCGGGGACCGCGTCTGCGCGCTCTCGCTCTCCGGGCTCGCCGTGGCGCGGGACGCTCTCGGG
ATGGACGACGACGCGCTCGTCGTCGCGCTCAAGGCCCCCGGCGTGTGGCGCGTCGATCCGGTGCTCGCCGCGGTGGGTGACCGCGTGGTCGCCATGGGC
GGCGCGTGCCGGCTGGCGCTGGGTGACGGGGAGGACACCTCGGCCGTCGAGGTCCACGAGCGCGGCGGCTGGACGCACTGCGGCGCGGTGCCGACCG
CGCTCCGGGAGTCCGCGGCGGCGGCAGCGACGTGGCTCTCGACGGCGGCGACGGATCAGAGAGTGTACGTCGCGGACAGAGCCACGGGGACGGCGAG
CTGGTTCGACCCGGCGAAGCAGCAGTGGGGACCCACCTCCCGCCTCCGCCCTGACGCCACCGTCTCCACATGGGGCCTCGCCGCCGGCCGCGCCGGCG
CCGAGAAGATCATCCTGTTCGGAGTGAAGCATGCAGACAGCCGAGTCGTCATCCGATCATGGGAGGTGGACGGCGACAGCCTCTCCCTGTCCCATGGCG
CGGCGGCGGCGCACGACACGATGCCGAGCGAGATGTCCGAGAGGCTGTTCCCGCACGGCGACGACGGCGAGGAGGAGACGTCGTCGCCGTCGATCGG
GGTGTGCGGCAACACCGCCGGTGGGTACGTGTACAATGCGGCCGTGCCGGCGACGGGCGCCGTCCTCTACGAGCTCCGGCGGGGCGGGGTGGAGGGCG
GCGGCGTGGAGCGGTGGGCGTGGGTTGCGTGCGCGCCGGTGGTGGCCGAAGCGGAGGCGTTGGGACGCGTCATCCTCGCGTGTTCGCCGGTGGGGCTG
CACGAGCTTGCGGACGAACGGCTTGCACACTGAACTAATAATGGCTATCGTGACATGTTGTCGATAGCCAGTTGAGCTTGTAATAAGCATATAGGGCTGTT
ACACTCTGAAAAATAAGTTTGGATACTCCCACCGGTTTTATAATAATTCTCGTTGTTTTGGACAATAATAACACGGCATATTCACTACATGACAAGTAAAAA
TCATGTGATTGGTTGACG>

TCONS_00010138; FPKM value: TC=12.9725, TT=2.13301, SC=2.6407, ST=8.5666.

CACATGACGAAGTGAGCGGGCTCTTTGTAGCCCTGGAAAGTCCCCGGGCCCTCAACCGCAGAGATGCGGCCGCGGAGACGCGGAAAGTCTCAGCGCTC
GCGCCCTCCTCTTCGCGAGGGGGCAGAGACACGTGTGGAGCCCTTTGGGCGCGAGCCGAGATCATAATTGGCCAATTACACAGTTATTCACTTGATCCAT
CTCCGGATTAACAGAACGGGGCTTACGATTGTGTGCAAAA>

TCONS_00032475; FPKM value: TC=14.6353, TT=2.65753, SC=109.671, ST=0.620943.

CATAAATGCAAGGACACATCATCGTAAGATTTGGTTGTGCCCCTATATAAACATGAGCAAAGGGGCACAACAATGAGCACAGTGAAACTGAAGAGGGCA
GATACATACAAACAAGAGCTCAGAGCTTGTTTCTTCAGTGATCTCTCCTAATGGCAGCTACCAAGAGGATGGCTTACTCCATCTCGGTGGTTTTCATGCTT
CTTTTGATCATCTCTTCCACCTTCTCATCCTGCTACGCAGCTAAAGATATGAAGGACCGAGAAGCAATAATACAGCGCCATCGCGAAGAGTGTAAGAAAAC

ATGCAGAGAACGTCATACTTACTGCCCGCCTGAGATGCCATGCTGCTGCATGAAGAACGCCCCGGCATCATCTCCCAGTAGTGTCAAGAATGAAAGCGGC
GA>

TCONS_00042245; FPKM value: TC=0.569332, TT=2.10755, SC=1.24878, ST=0.348106.

ATATCTCGTTTTTTTCTTTCTTCCTTGTCCATCCCTCTCTCTCAATCGTTTTTCTTTCTTTCTTCCTTAGCATCGAGCGTCCACGCGCCGAGCACCGGGTCCG
GTGGCGGCCTCTGACTCCATGCGTGAGTAGCGGCCACCGCCGGCCACCGGATCTGGCCTATGGGGCGCGGATCCGGCCACCACAGCCTCTTCCCCCACC
GCCGGCCGCTTCCTCCTCCGCCTCCTGACCGCCAGCTGACGCTGCCTCCCCCGCCTTCCGACCGCCGGCCGCCGGATCTGGCCTACAGGGCGCGGTTTCG
GCCACCGCGGCCTCTCCCCCCACCGTCGGCCACTTCCTCCTCCGCCTCCCGACCGCCGGCCGCCGCCGCCTCGCCCGCCTAACGACCGCCGGCCTCGAC
ACGTTTGGGGAACGCGGATCCGGCGCCTATCCCCCGACCGCCAGTCGCCTCCTCCTCCGCCACCCCACCGCCGCCTGCCAGATCCGACGACAGCTGCAC
CGCCAACCGTCGGATTCGGCGGCCTCTACCCCCGACTGCCGGCGTCGTCCACCGTCTCCTTCTTCACCCGCTAGCGCCGCCGCCATTTTCAACTCTTGTAG
GTTGTGCAAATTTTCATTGCCAAATTTCAGATTGGGTTATACTCACGGTTGCTTGTGTGGATTTGTAGTTGCTTGTGTGGATTTGTAGTTTATTACTCAAAGT
TGGTGTGGATTTGTTATGTATATTACTTGTGTGGATGTGCTTCATGTGTCAACATATCTGTCAATGAAATGCAAAGTTATTCTGTCATGATATCTTAAGATGTT
CTTCATGTCATGAGATATGGCGTGTGGGTTGAGAGCTGAGAGCTAGAGGCTGAGAGCTTTTGAATTTTTTTTGGTTGATTTTTCACACCTGTGACAGGTGG
TGAGTTGAACCTGTCAGAGGTGTTTGTCACCTTTGATGGGCTAAAAAG>

TCONS_00050051; FPKM value: TC=1.53373, TT=0.17095, SC=12.0855, ST=0.0166578.

GTCAGTTCAATTCAGTTCATCTTATCTGCCATGAAGGCCAAGGTTGCAGCAACAGCCCTGTTCCTGCTCCTTCTGACCTTTGGTGGTGAGGCCAAGATATG
CCATGATCCCAGCCAAACCTTCAAGGGGATGTGCCTCAGTAACAGCAACTGCATCTCCAGCTGCATCTCCGAGGACTAGACCGGCGGCTACTGCGGCGG
CGTCATAGACCGCAAGTGCATGTGCACCAAGGAGTGCGACGACGCGCCGCCGCCGCTGCCGTCGCTGCCGGCGCCGAAGAGCGGGCGGCGGCGTCCGC
CGCCACGGGCGCCGGGGGTGACGACGACGATCGAATGAGGACTGAAGAGAGGCTGTGTTGTGTGTGGTGTGTGGTGGCTATTCTGATTCATGTTCAATTT
AATTTTCGATCGGTCGAAGATAA>

TCONS_00078261; FPKM value: TC=40.9568, TT=69.5497, SC=19.6294, ST=136.762.

CGTCGCCCGAGGGCCGCATTTTATCGTGATCCATCACACACGTCCATGGACAATCCGACGGCGAGGCACTCGTCCACCTGTCCATCTTGGAACTAGTCGTT
GGATGATTACCTGGTAAAACAACCGCTTCTCCCTCCCGGCGACGCGGTGCCGCTGCACGAAGGGTCAACATAGCCGGCGCCGCTCTCGCGACGAAGGTG
GTCGGTGCCAGCCGGACCCAACCCACTGACGTGTTGGTCCCACTCGACGTGGCCCCCACGTGTCGGCGACCGTTGCCGTTGCGACTTCGTCACGACTTC
AATAGGCTACAGCTGTTGCTTAACCGACATGGTGGTATACTAGCCTTTCGGCTATAAAAACCTGGACCCTCCCATTGTTTTCGCCACACAGTTCATCGGCAT
CTTCTCACACTCTAGCTCACAGCTTCGCGGTTTCCACGCACTTGAAAACTCACGTTTCCAAGCCGGAAAACCATGGCTCGCTTCGCCGTAGTCGCCGCCA
TCGTCGCCCTCCTCGCGGTCACGGCCGCCGCGCAGGCCCCGGGAGCGGCCCCCGTCCCGGCGCCCAAGATGGCCCCGCTGCCCGCTCCCCCGGCGAGGT
CCCCGGCCACCGCCCCCGCGCCGGTCGCCACCCCGCCCACCGCCGCGTCGCCGTCCCCCATGGCGTCTCCACCGGCCCCGCCCACTGACGCCCCCGCCG
CCAATGCCCCCTCCGCCGTCACCCCGTCCGCGGTCACCCCCTCCTCCGTCTCCGCCCCGACCGGCGCCCCCGCTGCCTCCTCCACGTACACCGCCACCGC
CAGCTTCGTTGCCGTCGCCGGCGCGGTCGCCGCCGCCATCGTGTTCTAGATGGATGGATGGATGATTTGATCGACGCGTTGTTTATACGGCGAGATTCTTT
TTTGTGATTCTGTTCTATTTACAGACAGCGAGACACTGATATTCAGATGTTTACACTTGTCATGTTTGATGAGATGATTATCGATGGAATATATTCTTTCATTT
ATTATTACCATACATGGAGTATTGTGCAATTTTTTTCCATTTTCTTATATTATCTGTATCGCTCACGCGTAAACTATTACTGCCGATCTGATCGGGCCAACCTAC
AAGAGTGGCTGTCACATGCATGATAGTCTAGATAGCTTTTTTTCCCTTTGCTTTACCG>

TCONS_00135011; FPKM value: TC= 0.00451993, TT=0.408014, SC=0.229268, ST=0.704973.

CTCTTATTCCATTACATGCTCGATCAGTAGTGAAAGAAATCAGAGGTTAGAAGTTTATACAGTAGTGCATCAGCTTACCACAAAGGTGGAATGGCTCTAGG
AGATGTGAAAATGTGAATAGTGAACATGAATGCCATAATGTAGGAATGAGGTACATCGAGGAGGATTAGAGGAGACTCAATGTAACAACCCAGCCGAGC
AATAATAGTCCCACATGTAGAGTTTAGATTGGTTGAGCACCAGCACATAAACCCGTGCGTTCTAACTCTGTATGGATTAACTCTTTTACATATAGCCAGAAT
AGTAGAAGTATATAAATTCCTCGAAAGAGCTGAGGTGTGGGCTTTTCAGAAACTAAGATAGCAGCACCGACACTAACTTTAGTAGAGAGGTGAAGAGCC
TGTAAGTTCTGTCATTTGTGATGAAGAAGAAATGATCAGATTCTCTGTGAGCTCACAAAACCAAAATGAGCAAGTACTTCCGGACAGTATGCTCATGCAC
CTACAGCACTTCATTTGAACTTGTTCCACACCTTGTTTAATAGAGTTTCTTATTCTACTAGAAAGAGAAACCATTGTAAAACATCTATATTTTCTCACATAGG
ATCCCCCAAGGAAGAGACTAATATTAGAACAGAACAATCATGAAAGTCTTCCTCTTCAAAAAATATAAGGGGAGGGGGGTGTCATTAGGATGTGACAAAA
TTTGGGGAAAAAATACGATGCCATGATAACAGAAAAGAGGAATAATGTTTTTATTTTGACGGAAAAAGAGGAATAATGTTGGTAATACATGATAATCATTT
CAGAAAAAAAGGGAATGTGTGCAATACATGTGCATATGTGATTATCATTGACCCCTTGAACTAGTCAAATCTAACACCAACTTTTATATATGAATTTGAACA
TAACCTATGTCTAGATTTGTATACAAAAGTTAATTTATTTTGGGATGGACGAAGTATATCAATATTGCGAATGTTTTCAATCAGAGAAGTGGACAGATATGAA
CATTTTGGTTTACACATAAGAACAACTTATCATGTCGTGGTCAATTCATGTTTGATGTCTAGGTCTTTTGTCTTCTGGAACAAATTTTTATTCCTGGTACGAA
ATAGTATCAAATAATTATAATCTTGAAACCATCGAATCGTGTGAATAAAAAAAAAACCAGAACTGCAGAAACACACAACGGACATGTCTCAAATTTCATAAG
TTATTGAACAGAGGAGTAAATTATAAATATGATTGATGATTTCTCAAACCAACAGGTGGTGAATGTAAAAGGTGACCATAACTCTCAGGGAAAAAAAAGC
ATTCACCAACCTACCACTTATTCATATCAGTTATAAATAACTAAACACCTGCCTTTCATTTTTATCAGTGCCACTTCGTTGTTTTCTTCCTTTCTACAGTTACA
AGAACTAAACTTGAAGCAGTTGCCACACATCAGTTAAAGAGCGAGGAGTAATCTCTACCACAAAATGCATGGGTATGCAACTGTGAGCTTAATTCATTGG
TTCACACCCTCATTTATCTGATCTGTTGTAGGGATGGGATGCATTGAGGAAGATAAGAAAAGGCACAACGATAATACCGGGAGTGTTTCAGCATCGATCGG
TCCTGATTCCTGACCTTCTTTCAGCATGAAGTGCGACACTTGCATCTCACACTAGGACAGATGTCATGACCTCAGACCATGACGGAATAACTACAGCCGG
GAAGATAGCCGGGCAGCGTCGGCTCCTGGAGAAGGGATGTAGATTCAGGCTAAGGAGAAGAACATTGCTGGGAGAGAGAGATTAGATTAATT>
