# Supplementary material for: Transcriptome changes in rice (Oryza sativa L.) in response to high night temperature stress at the early milky stage
Source: BMC Genomics. 2015 Jan 23;16(1):18. doi: 10.1186/s12864-015-1222-0 (PMC4369907; doi:10.1186/s12864-015-1222-0)
Supplement: Additional file 6: — The differentially expressed HTRTs without reference genes and/ or functional annotation. [file 12864_2015_1222_MOESM6_ESM.pdf]

Additional file 6

The differentially expressed HTRTs without reference genes and/ or functional annotation.

Transcripts Name	Size (bp)	Reference Gene in GenBank database
TCONS_00003660	650	osa:4324216
TCONS_00052789	1017	osa:4329572
TCONS_00053233	749	osa:4325996
TCONS_00075473	1532	osa:4334845
TCONS_00108299	991	sbi:SORBI_09g013380
TCONS_00116758	915	osa:4340023
TCONS_00140366	1485	osa:4345515
TCONS_00152703	1697	osa:4346638
TCONS_00010138	238	
TCONS_00032475	403	
TCONS_00042245	957	
TCONS_00050051	421	
TCONS_00078261	1167	
TCONS_00135011	1811	
