# Supplementary material for: Transcriptome changes in rice (Oryza sativa L.) in response to high night temperature stress at the early milky stage
Source: BMC Genomics. 2015 Jan 23;16(1):18. doi: 10.1186/s12864-015-1222-0 (PMC4369907; doi:10.1186/s12864-015-1222-0)
Supplement: Additional file 7: — The RT-qPCR primers for the 12 selected unigenes. [file 12864_2015_1222_MOESM7_ESM.pdf]

Additional file 7

The RT-qPCR primers for the 12 selected unigenes. Primer sequences were designed on exon-exon boundaries using the online primer software primer-BLAST in NCBI according to the base sequences of the assembled transcripts.

Transcript name	Forward primer 5′–3′	Sense primer 5′–3′
TCONS_00050124	CCTCAAAGGGAAGCCCACAG	GGCACACGAAGAGTTGGGAG
TCONS_00134261	GAGCATGGTGTGGCAGGTAT	GTCGAGCGACCATGTCTTGA
TCONS_00070653	AGCCATGGATAACGCCTACA	CGAGGATCTTCCTCCTGACG
TCONS_00055988	GGTGGCCGGAAAGTTACAAG	ACCTCGGACATGGTGTACTG
TCONS_00100761	CCACTCGTACACCGAGTTCC	GTGCGGCGTACGAAAAACAG
TCONS_00072597	TCTTCTCCTCCAAGATTGCCAC	AATTAGTACCCGCCGGTGAA
TCONS_00016258	TTCTTACCTGGGGCATCCTGT	GGGGCAAATCCGAATGTCTC
TCONS_00119170	CAGAGGTACAGCGACAACAC	CTCTCAACTCCGGCGAACTC
TCONS_00078028	AGTCAAACTGAGCTGCCTGA	GCCGAAGTACGGGAACAGC
TCONS_00090884	ACCGACTTCCACACCTACTC	TTCCGTGGCGGTGAAGTTG
TCONS_00091734	AAGGCTGCTACGTGATGGAC	ATGCAGTTCTTCATCACGCTC
TCONS_00145395	CACTCCTTCTTCTTGCGACCT	GCTGGTCGTCCCCAGAATAA
TCONS_00054381	GACCAGTCGAACACCACCG	AGGGTACGCCTTGTCCATTC
TCONS_00002095	CGTGGTCGTCTCTATGCTCTT	TCCCCAGTCAGATTGCATCG
TCONS_00058465	GATATGGCTCGGGACGTTCG	GTTCAAACGCGCAGGATCAG
TCONS_00130099	CAACTACGGGACTGCCAAGA	GCAGATGAAGAGCACCACGA
TCONS_00144852	CATGAACACATGGTCGGAGA	CTCAGGACGGAGAAGAAGTCC
TCONS_00053041	AGCAACACAATCACCACGAC	GACGTCGTACCCTTCCTCCT
TCONS_00001933	CCTCGGGCCATCTACTGAAC	CGGGAGCATGGTTACTCGAC
TCONS_00115693	CCGGCAAGCACTACTTCATC	AAAGCACCAGTTCCCTCTGT
TCONS_00032876	GATGCGAAGGTTGGGTTTGG	TGGCTTGCTCATCGTCTTCC
